# Novel Translational and Phosphorylation Modification Regulation Mechanisms of Tomato (*Solanum lycopersicum*) Fruit Ripening Revealed by Integrative Proteomics and Phosphoproteomics

**DOI:** 10.3390/ijms222111782

**Published:** 2021-10-29

**Authors:** Qiaoli Xie, Yanling Tian, Zongli Hu, Lincheng Zhang, Boyan Tang, Yunshu Wang, Jing Li, Guoping Chen

**Affiliations:** 1 Key Laboratory of Biorheological Science and Technology Ministry of Education, Chongqing University, Chongqing 400044, China; Tianna0816@163.com (Y.T.); huzongli71@163.com (Z.H.); zhanglc201312@163.com (L.Z.); atangboyan@126.com (B.T.); wangyunshu@cqu.edu.cn (Y.W.); micy180605@163.com (J.L.); 2 Bioengineering College, Chongqing University, Chongqing 400044, China

**Keywords:** proteomics, phosphoproteomics, fruit-ripening, *rin*, tomato (*Solanum lycopersicum*)

## Abstract

The tomato is a research model for fruit-ripening, however, its fruit-ripening mechanism still needs more extensive and in-depth exploration. Here, using TMT and LC-MS, the proteome and phosphoproteome of AC^++^ (wild type) and *rin* (ripening-inhibitor) mutant fruits were studied to investigate the translation and post-translational regulation mechanisms of tomato fruit-ripening. A total of 6141 proteins and 4011 phosphorylation sites contained quantitative information. One-hundred proteins were identified in both omics’ profiles, which were mainly found in ethylene biosynthesis and signal transduction, photosynthesis regulation, carotenoid and flavonoid biosynthesis, chlorophyll degradation, ribosomal subunit expression changes, MAPK pathway, transcription factors and kinases. The affected protein levels were correlated with their corresponding gene transcript levels, such as NAC-NOR, MADS-RIN, IMA, TAGL1, MADS-MC and TDR4. Changes in the phosphorylation levels of NAC-NOR and IMA were involved in the regulation of tomato fruit-ripening. Although photosynthesis was inhibited, there were diverse primary and secondary metabolic pathways, such as glycolysis, fatty acid metabolism, vitamin metabolism and isoprenoid biosynthesis, regulated by phosphorylation. These data constitute a map of protein—protein phosphorylation in the regulation of tomato fruit-ripening, which lays the foundation for future in-depth study of the sophisticated molecular mechanisms of fruit-ripening and provide guidance for molecular breeding.

## 1. Introduction

The ripening of fleshy fruits leads to many changes in morphology, colour, nutritional quality, fragrance and the maturing of seeds, representing a unique coordination of developmental and biochemical pathways [1]. During ripening, the fruit becomes soft, its colour becomes attractive, and volatile compounds and sugars accumulate, all of which, ultimately, facilitates the spread of seeds [2]. As fruit is a food rich in nutrients, such as vitamins, minerals, antioxidants and fibre, the investigation of fruit-ripening is of great significance to basic plant biology, horticulture and food-nutrition engineering.

As the simplest chemical hormone of plants, ethylene is important for the ripening of menopausal fruits, especially the tomato [3]. Most of the time, ethylene regulates fruit development by initiating multiple signal transduction pathways through ethylene-receptor binding and downstream signal transduction [4]. Sometimes, however, ethylene alone is not enough, showing that ethylene-independent pathways play a role in fruit-ripening [1]. Over the years, studies of tomato ripening mutants have provided a wealth of information on the mechanism of tomato fruit ripening [5,6,7]. Among these mutants, *rin* is necessary for both ethylene-independent and -dependent pathways [8,9,10]. LeMADS-RIN has been cloned and encodes a MADS domain transcription factor that inhibited all ripening phenomena, including ethylene production, respiratory climacteric, production of flavour compounds, carotenoid accumulation and softening [5]. This demonstration that *rin* fruits cannot ripen suggests that RIN is not only critical in the regulation of fruit-ripening, but also contains genetic regulatory components that trigger ripening-related menopausal respiration and ethylene biosynthesis [11]. In another study, F1 plants were developed between wild-type and *rin* plants, and their fruits had longer shelf lives and were of better quality, an important goal in tomato breeding [12].

To study RIN at the protein level, Zhu et al. [13] obtained a polyclonal antibody specific to RIN and found that, during fruit-ripening, RIN mRNA increased, which coincided with RIN protein accumulation. Subsequently, they obtained RIN that can bind to DNA through an optimal expression and purification system [14]. RIN proteins, accumulate only in ripe fruit and bind to the cis element of *LeACS2* [15]. The *rin*-mutated proteins that accumulate in mutant fruits has also shown DNA-binding activity, though it lost its transcriptional activation, thus inhibiting the ripening of the mutant fruits [15]. By ChIP and qPCR, the analysis of a potential binding site (CArG-box site) of RIN, in the promoters of the ripening-induced genes *LeACS4*, *TBG4*, *LeACS2*, *PSY1*, *LeMAN4*, *LeEXP1*, *LeACO1*, *ETR3*, *PG* and *INV,* showed that, in the promoters of *PG, LeACS4, LeACS2*, *LeMAN4, LeEXP1*, *TBG4* and *RIN* itself, CArG boxes were enriched nearly 5- to 20-fold. Virus-induced gene silencing (VIGS) analysis showed that the ripening was inhibited because *LeRIN* silencing inhibited the expression of *LeACO1, LeACS2,* and *LeACS4* [16]. *Nor* (non-ripening), *rin* and *Nr* (never-ripe) mutations suppress ethylene signalling and ripening. A systematic analysis of the metabolomics, transcriptomics and proteomics levels of *NOR*, *RIN*, and *Nr* during development and ripening revealed that, in the early stage of ripening, the expression of *Nor*, *rin* and *Nr* transcripts correlate less with the expression of their corresponding protein. This suggests that post-transcriptional regulation may play crucial roles in the corresponding stages, as the correlation was more pronounced at later stages. Furthermore, the correlation between a specific metabolite and its ripening-associated transcripts (such as cell-wall metabolites, organic acids and sugars) is very strong, highlighting the significance of metabolic regulation during fruit-ripening [17]. RIN can adjust the expression of the GRAS-family transcription factors, CNR and TDR4, directly [10]. Another five genes *ADH2, PGK*, *PNA*, *E8* and *TomloxC,* were identified as new direct targets of RIN by ChIP [18]. A total of 241 direct-target genes of RIN were evaluated by ChIP-chip [19]. The ethylene-biosynthesis gene, *LeACO4,* and the cell-wall degradation gene, *alpha-Gal,* were directly adjusted by RIN [20]. In addition, RIN forms complexes with FUL1 and FUL2 that can regulate ripening genes [21]. The initiation of fruit-ripening was not inhibited in *RIN* knockout (CRISPR/Cas9 method) mutant, and the fruits were moderately red [22]. Furthermore, the inactivation of *rin* mutant alleles only restored the ripening ability of some plants. RIN, therefore, is a gain-of-function mutation that generates a new protein to inhibit ripening [23]. Small heat shock proteins (sHSPs) are regulated by MADS-RIN in an ethylene-dependent manner [24]. Recent studies have found that RIN-MC encodes a chimeric protein that leads to the *rin* phenotype [25]. RIN can regulate SAUR69 in the ripening transformation of tomatoes by regulating their ethylene sensitivity [26]. In a study of epigenetic and non-coding RNA regulation, whole-genome bisulfite sequencing showed that binding sites of RIN are usually located in the demethylation region of many ripening-genes’ promoters, and that the binding and demethylation occur simultaneously, indicating that the dynamic changes of the epigenome can regulate fruit-ripening [27]. ChIP and EMSA confirmed RIN can directly bind to the promoter of MIR172a to regulate tomato fruit-ripening [28]. For LncRNAs, RIN can directly target 187 lncRNAs. In addition, tomato fruits, in which lncRNA2155 was knocked out through use of CRISPR/Cas9, showed delayed ripening [29]. These results proved that the regulatory network by which RIN regulates fruit-ripening is quite complex at the level of transcription, metabolism and epigenetics.

The function of a gene is ultimately demonstrated by its protein. Due to post-transcriptional and post-translational changes, the correlation between transcript and protein levels is poor [30]. Proteomics can provide a powerful way to discover proteins related to fruit-ripening and to elucidate key pathways of fruit-ripening. Using iTRAQ combined with MS/MS, proteome analysis of tomato pericarp at different ripening stages (39, 42 and 52 DAP) of wild-type and *Nor*, *rin* and *Nr* were carried out, and 158 differentially expressed proteins were identified [17]. The proteins of wild-type and *rin* fruits were separated by 2D gel electrophoresis and Q-TOF MS/MS, and 41 protein spots, representing 35 independent genes were successfully identified [18].

Post-translational modification (PTM) is the post-translational chemical modification of proteins, including ubiquitination, phosphorylation, acetylation, methylation, glycosylation, crotonylation, succinylation, propionate diacylation, dihydroxyisobutylacylation and lactic acidation, etc. It can regulate the activity, localization, folding, and interaction of proteins with other biological macromolecules (including proteins, nucleic acids, lipids, etc.). Phosphorylation is a widely used type of post-translational modification, which refers to the process of transferring the phosphate group of ATP to the amino acid residues (threonine, tyrosine, serine) of the substrate protein by a protein kinase. More than 30% of proteins in cells are phosphorylated. Phosphorylation participates in various physiological and pathological processes, such as cell development, differentiation, apoptosis, plant growth, stress resistance and other areas. Therefore, phosphorylation modification is the most basic, most common and most important regulating mechanism for controlling proteins’ vitality and function [31].

Therefore, phosphorylation may have a regulatory mechanism to coordinate fruit-ripening, and may have effects in the network of RIN-regulated fruit-ripening.Fully understanding *rin* could shed light on some new genetic regulatory mechanisms associated with fruit-ripening. Here, integrative proteomics and phosphoproteomics of wild-type and *rin* tomato fruit were studied. The data of this study will explicitly reveal a large number of differentially expressed proteins and phosphorylation sites, which will help infer the phosphorylation regulation mechanism during fruit ripening and coordinated development. The results of this study will not only enrich the knowledge of the translation and post-translational regulatory network of fruit-ripening, but also provide a new perspective for improving tomato fruit quality.

## 2. Results

### 2.1. Proteomic and Phosphoproteomic Profiling

**Figure 1:**
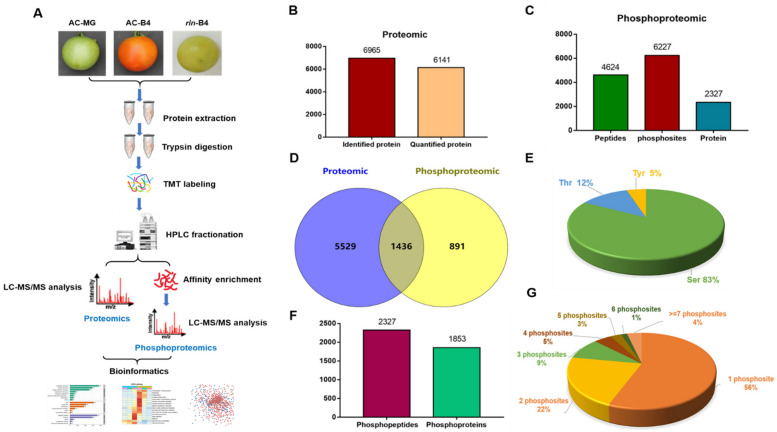
Summary of proteome and phosphoproteome data. (**A**) Workflow of the integrated proteome and phosphoproteome experiments. (**B**, **C**) Statistical results of proteome and phosphoproteome identification. (**D**) Venn diagrams of differentially expressed proteins and different phosphorylated proteins identified in two omics. (**E**) Distribution of identified phosphorylated serine, threonine and tyrosine residues in the phosphoproteome. (**F**) Statistics of differentially expressed phosphorylated proteins and corresponding peptides in the phosphoproteome. (**G**) Proportion of number of phosphorylation sites per protein in the phosphoproteome.

To explore the roles of protein expression and protein phosphorylation in tomato fruit-ripening, the proteome and phosphoproteome of the pericarp of AC^++^ fruits at MG and B+4 stages and *rin* fruits at B+4 stage was profiled by using the quantitative tandem mass tag (TMT) proteomic and phosphoproteomic approach (Figure 1A). A total of 6965 proteins were identified in the proteome, of which 6141 were quantified (Figure 1B and Figure S1). A total of 4624 unique phosphopeptides spanning 2327 proteins with 6227 sites of phosphorylation were identified (Figure 1C and Figure S2), among which 4624 peptides in 1996 proteins were accurately quantified (Figure 1C). There were 1436 proteins shared by the two omics (Figure 1D). Combining the two data sets, we have completely identified 7856 proteins from tomato fruits (Figure 1D). Of the 4842 nonredundant phosphorylation sites, the quantities of phosphoserine (pSer), phosphothreonine (pThr) and phosphotyrosine (pTyr) were 4017 (83%), 592 (12%), and 233 (5%), respectively (Figure 1E). The phosphopeptides and their corresponding phosphoproteins showed that 2327 phosphopeptides and 1853 phosphoproteins were identified in all the three fruit stages (Figure 1F).

### 2.2. Characteristics of the Phosphorylation Sites

The number of phosphopeptides in a particular phosphoprotein varies greatly. In this study, among the 2327 phosphoproteins, 1306 (56.1%) had only one phosphorylated site, 505 (21.7%) had two phosphorylated sites, and the rest 516 (22.2%) had three or more phosphorylated sites. It is noteworthy that approximately 4% (90 proteins) of the identified phosphorylation proteins had at least seven phosphorylation sites (Figure 1G). There were five proteins with more than 15 phosphorylation sites, and 12 proteins with more than 10 phosphorylation sites. There were 22 phosphorylation sites (16Ser + 6Tyr) in the splicing-factor protein SFRS4 (K4DAU1), 21 phosphorylation sites (15Ser + 6Thr) in the RPM1-interacting protein 4 (K4DHL7) and 18 phosphorylation sites (13Ser + 5Tyr) in the uncharacterized protein (K4BBY2).

### 2.3. Identification of Differentially Expressed Proteins (DEPs) and Phosphoproteins (DEPPs) at Different Stages

At different stages of fruit-ripening, there were significant differences in the number of differentially expressed proteins (DEPs) and differentially modified phosphorylated proteins (DEPPs), among which the AC-B4/AC-MG group had the most DEPs and DEPPs, while the rin-B4/AC-MG group had the least (Tables S1 and S2). In the three different stages, the DEPs and DEPPs were mainly up-regulated in AC-B4/AC-MG, and the up-regulated proteins accounted for 32.82% and 25.39% of the total DEPs and DEPPs, respectively. The DEPs and DEPPs in AC-B4/AC-MG were mainly down-regulated, which accounted for 35.42% and 53.52% of the total DEPs and DEPPs, respectively. The above results show that the greatest change occurred in the period from MG to B4 in the fruit ripening process.

**Figure 2:**
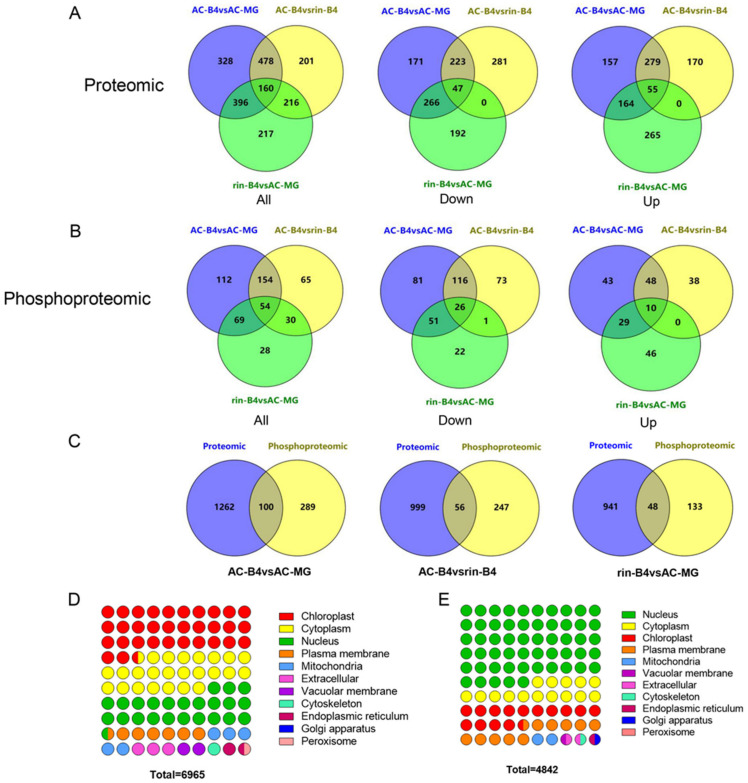
The overlap and subcellular localization of the differentially expressed proteins and phosphoproteins. Overlap of the differentially expressed proteins (DEPs) and phosphoproteins (DEPPs) in Venn diagrams with different comparisons. (**A**) DEPs; (**B**) DEPPs; (**C**) DEPs and DEPPs of two omics. The subcellular localization of the fruit proteome protein (**D**) and phosphoproteins (**E**) predicated by GO annotation.

The analysis of DEPs found that 160 proteins in the three groups had significant changes, while 328, 201 and 217 proteins only had significant changes in AC-B4/AC-MG, AC-B4/rin-B4 and rin-B4/AC-MG, respectively (Figure 2A). By analysing the DEPPs, it was found that, in all groups, 54 phosphorylated proteins had significant changes, while 112, 65 and 28 phosphoproteins changed significantly only in AC-B4/AC-MG, AC-B4/rin-B4 and rin-B4/AC-MG, respectively (Figure 2B). Among the up-regulated proteins, 55 proteins in the three groups were co-up-regulated, while only 10 proteins in the three groups were co-up-regulated for the phosphorylated proteins. Among the downregulated proteins, 47 proteins were co-down-regulated in the groups, while 26 co-down-regulated proteins for phosphoproteins were in the three groups. The protein distribution between the two omics was further analysed. The results showed that the AC-B4/AC-MG group had the greatest number of co-expressed proteins, 100, while the rin-B4/AC-MG group had the least, 48, and the AC-B4/rin-B4 group, with 56 co-expression proteins, was in the middle (Figure 2C).

In the proteomic profile, ACO3, LeEXP1, GSH1 and LeSRK2C were significantly increased in all groups (Table S1). Similarly, in the phosphoproteomic profile, 40S ribosomal protein S6 (K4CME5), ACO3 and Auxin repressed/dormancy associated protein (Q0PY39) were significantly increased in all groups (Table S2). This shows that variations in ethylene biosynthesis, the assembly of ribosomes, cell wall development, ABA signaling, auxin response and glutamate-cysteine ligase have significant effects in tomato fruit-ripening. In the proteomic profile, CAB1B (LHCB1), LHCB4, LHCB5, LHCA1, LHCA2 (CAB7), HCA3 (CAB8) and LHCA4, psbB, psaC, PSBR, PsbQ and psaA were significantly decreased in all groups (Table S1). Analogously, in the phosphoproteomic profile, LeSRK2C, cysteine proteinase inhibitor (4BJE4), phosphoinositide phospholipase C (K4C1V6 and K4C5V3), LHCB1 and protein kinase MRLK1 (K4CS51) were dramatically decreased in all groups (Table S2). These proteins are closely related to ABA signal transduction, chlorophyll metabolism and photosynthesis, indicating that these pathways play a vital role in tomato fruit-ripening.

Additionally, in the proteomic profile, carbonic anhydrase (ca3), lipoxygenase (K4ASM0), long-chain-alcohol oxidase (K4CI54), alcohol acyl transferase (Q6QLX4), ferredoxin (K4D4V2), glycosyltransferase (K4B2Z9 and K4CWS6), ribokinase (K4CGP0), malic enzyme (K4DBT1), beta-galactosidase (K4DBX2), temperature-induced lipocalin (Q38JD4) were up-regulated, and secretory carrier-associated membrane protein (K4CFZ4), PR10 protein (K4CWC5), GAR1 ribonucleoprotein complex subunit 1 (K4D296), petA (Q2MI87), Cytochrome f (Q6J1L7), GDP-mannose pyrophosphorylase (Q84P53), Gamma aminobutyrate transaminase 2, GABA-TP2, Serine hydroxy methyltransferase (K4BCV4), and In the phosphoproteomic profile, uncharacterized protein (K4AT42, K4AYW0, K4B1F9, K4B3R1, K4B988) and coatomer subunit beta (K4DD78) were down-regulated.

### 2.4. Characteristics of the Cellular Localization

Subcellular localization analysis showed that in the proteomic profile, the proportion of proteins located in the chloroplast is the largest (2255), followed by the cytoplasm (1700) and nucleus (1616), while in the phosphoproteomic profile, 255, 401, 416 and 1096 of the phosphorylation proteins were positioned at the plasma membrane, chloroplast, cytoplasm and nucleus, respectively. The total number of phosphorylated proteins in the remaining compartments was only 3.26% (Figure 2D). The distribution patterns of other cell components were similar in the two profiles, with a larger proportion assigned to the plasma membrane, followed by mitochondria, except for the cytoplasm, nucleus and chloroplasts (Figure 2D,E and Tables S3 and S4). It can be seen that many chloroplast-related proteins have been determined in the proteomic profile, and nuclear-related proteins have been identified in the phosphoproteomic profile, which proves that phosphorylation regulation in the nucleus is an important part of tomato fruit-ripening. It is worth noting that the ethylene synthesis pathway, MAPK pathway, auxin, transcription factors and ribosome-related proteins were also found in the phosphoproteomic profile.

### 2.5. Motif Analysis of Phosphorylated Peptides

**Figure 3:**
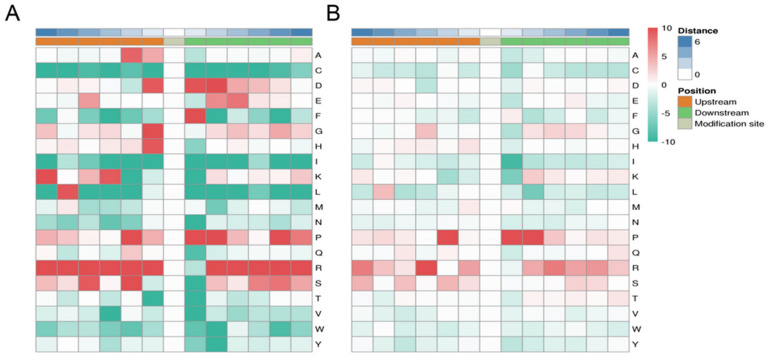
Analysis of phosphorylation sites. The motif enrichment heatmap of upstream and downstream amino acids of all identified S (**A**) and T (**B**) modification sites. Red indicates that the amino acid was significantly enriched near the modification site, and green indicates that the amino acid was significantly reduced near the modification site.

The MoMo software (version 5.0.2) [32] was used to identify phosphorylated lysine flanking motifs. Fifty phosphorylation motifs were found in 4080 peptides, including 38 pSer, eight pThr and four pTyr motifs (Figure S3A, Table S5. Among the 38 pSer motifs, motifs [RxxS], [GS] and [SP] accounted for the highest proportions of peptides, at 207, 227 and 342, respectively. In the eight pThr motifs, motifs [PxTP] and [TP] accounted for the highest proportion of peptides, at 78 and 97, respectively. In the four pTyr motifs, motifs [RxxxY] and [SxxxxY] accounted for the highest proportion of peptides, at 41 and 37, respectively (Figure S3B, Table S5). The heat map of amino acids around the phosphorylation site shows that arginine (R) and proline (P) were significantly expressed downstream and upstream of T and S. Other residues, such as aspartate (D) and phenylalanine (F), were frequently presented at position +1, and aspartate (D), glycine (G) and histidine (H) were frequently presented at position -1. The occurrence of cystine (C), valine (V), isoleucine (I), leucine (L), threonine (T), tyrosine (Y) asparagine (N) and tryptophan (W) was significantly reduced near S and T. Lysine(K), valine (V), threonine (T), serine (S) and asparagine (N) at the +1 and +2 positions were occurred least. Cystine (C), isoleucine (I) and leucine (L) at the +1 through -6 positions were occurred least (Figure 3A,B).

### 2.6. GO Term and Enrichment Analysis of DEPs and DEPPs

**Figure 4:**
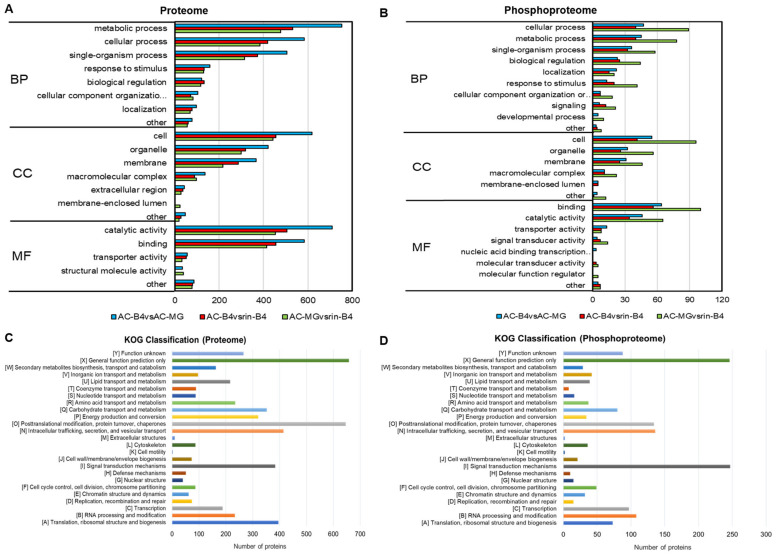
Top 20 GO categories and KOG classification assigned to the DEPs and DEPPs. Top 20 GO categories of the DEPs (**A**) and DEPPs (**B**). Top 20 KOG classification assigned to the DEPs (**C**) and DEPPs (**D**). BP, biological process; MF, molecular function; CC, cellular component.

GO is used to express the various attributes of genes and gene products. Our results show that, in the two omics related to fruit-ripening, the molecular function module mainly included transporter activity, catalytic activity, binding, molecular function regulators, antioxidant activity, signal-transducer activity, nucleic acid binding transcription-factor activity and structural molecule activity. Binding accounts for the largest proportion, close to 50%, followed by proteins with catalytic activity functions. In the cell components module, the distribution of protein functions in the two omics is also similar, mainly including organelles, cells, membrane-enclosed lumen, membranes, macromolecular complexes and other cell components. Cells account for the largest proportion, followed by membranes and macromolecular complexes. In the biological process module, the two omics mainly concentrated on single organism processes, cellular processes, responses to stimuli, metabolic processes and localization (Figure 4 and Table S6 and S7).

Notably, the biological process distributions of the two omics are different. In the proteome, metabolic processes occupy a major position, followed by single-organism processes. While in the phosphoproteome, cellular processes account for the largest proportion of processes, followed by metabolic processes and single-organism processes (Figure 4B and Table S7). The same trend was also found in further pairwise s of the difference proteins in the three comparison groups. AC-B4/AC-MG group had more DEPs than AC-B4/rin-B4 and rin-B4/AC-MG groups, while rin-B4/AC-MG group had the most DEPPs. It can be seen that in the fruit-development process, DEPPs in rin-B4/AC-MG group and DEPs at AC-B4/AC-MG group were the most (Figure 4). In the proteome, GO classification protein list of AC-B4/AC-MG and AC-B4/rin-B4 found that, during fruit-ripening, E4, Ca3, cell wall-related proteins PG2, Cel2, LeEXP1, MAN4, ethylene synthesis-related ACO1, ACO3, ACO4, ACO5, the synthesis of linoleic acid flavour substance LOX1.2, the related enzymes of pigment synthesis, CHS2, CRTISO, PSY1 and ZDS, the cellular ribonuclease RNALX and the phosphokinases LeSRK2C and LeCDPK2, were all significantly changed. In the phosphoproteome, PSBR, K4CLP5, 40S ribosomal protein S6, ACO3, LeGAD2, Serine/threonine-protein phosphatase, PsbQ, psbH, Phosphoglycerate kinase, sucrose synthase, LHA1, S-acyltransferase, LeCDPK2, CYP, MRLK1, LET12, MPK1, Phosphoinositide phospholipase C, ACO1, Non-specific serine/threonine protein kinase, LeSRK2C, LeCCH, CDKC, PURA, Chlorophyll a-b binding protein and ETR4 were all obviously changed in different comparison groups. These results indicate that cell-wall metabolism, ethylene synthesis, flavour compound synthesis, pigment synthesis, ribonuclease and phosphokinase all play essential roles in fruit-ripening and were included in the regulation of phosphorylation.

KOG functional classification statistics for differentially expressed proteins were performed through database analysis. As seen from Figure 4, the results of KOG analysis are mainly divided into 25 functional clusters. The functions of differential proteins in the proteomic profile were mainly protein turnover, intracellular trafficking, translation, post-translational modification, vesicular transport, secretion and chaperones, biogenesis and signal transduction mechanisms and ribosomal structure (Figure 4C). In the phosphoproteome, the classification of proteins corresponding to phosphorylation differences was also mainly concentrated in chaperones, protein turnover, signal transduction mechanisms, vesicular transport, post-translational modification, RNA processing and modification, secretion and intracellular trafficking, and transcription clusters (Figure 4D).

GO enrichment analysis was performed according to the annotations of all identified proteins. The bubble chart shows the top 20 categories with the most significant enrichment.

**Figure 5:**
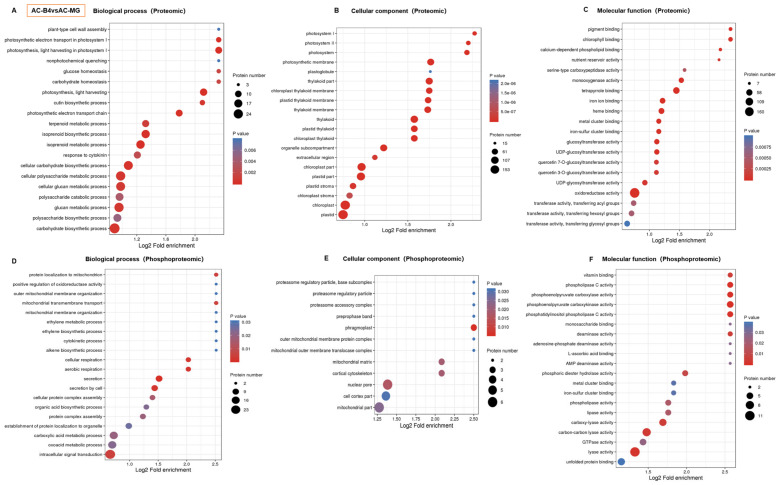
Bubble charts of enrichment distributions of DEPs and DEPPs in GO functional classification (AC-B4 vs. AC-MG). (**A**–**C**) bubble charts of enrichment distributions of DEPs (proteome) in GO functional classification; (**D**–**F**) bubble charts of enrichment distributions of DEPPs (phosphoproteome) in GO functional classification.

In the proteome, during the fruit-ripening process, the DEPs were mainly enriched in the processes of photosynthesis, carbohydrate synthesis, sugar metabolism, keratin synthesis, cell wall assembly, chloroplast synthesis, redox reaction and the anabolism of pigments (Figure 5A–C, Figures S4A–C and S5A–C). Contrarily, in the phosphoproteome, during fruit-ripening, phosphorylated differential proteins were mainly involved in cell secretion, cell respiration, intracellular signal transduction, mitochondrial transmembrane transport, mitochondrial protein localization, protein complex assembly, carboxylic acid metabolism, organic acid biosynthesis, ethylene synthesis and metabolism, gluconeogenesis, proteasome regulation particles, proteasome auxiliary complex and vitamin synthesis (Figure 5D–F, Figures S4D–F and S5D–F).

### 2.7. KEGG and Domain Enrichment Analysis of DEPs and DEPPs 

**Figure 6:**
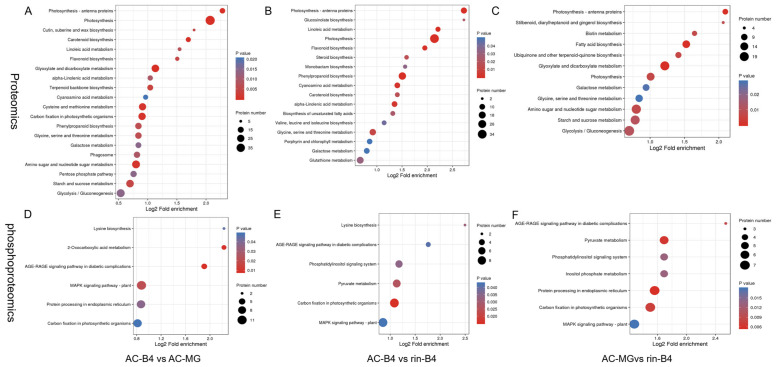
Bubble charts of enrichment distributions of DEPs and DEPPs in KEGG pathways. (**A**–**C**) Bubble charts of enrichment distributions of DEPs (proteome) in the KEGG pathways of different comparison groups; (**D**–**F**) Bubble charts of enrichment distributions of DEPPs (phosphoproteome) in KEGG pathways of different comparison groups.

KEGG pathway annotation and enrichment analysis showed that, in the proteomic, photosynthesis, photosynthesis-antenna proteins, glycine, serine, threonine metabolism and galactose metabolism are most changed pathways in the fruit ripening process. Carotenoid biosynthesis, phenylpropanoid biosynthesis, alpha-Linolenic acid and linoleic acid metabolism, flavonoid biosynthesis and glutathione metabolism are important regulatatory pathways in fruit ripening. Glyoxylate-and-dicarboxylate metabolism and starch-and-sucrose metabolism are important pathways in the gradual ripening from mature green fruit. Moreover, the cutin biosynthesis, terpenoid backbone biosynthesis, unsaturated fatty acids and fatty acid biosynthesis pathways were also enriched (Figure 6A,B).

In the phosphoproteome, the MAPK signalling pathway, carbon fixation in photosynthetic organisms and AGE-RAGE signalling pathways were found in all three groups (Figure 6D–F). Lysine biosynthesis presented in AC-B4/AC-MG and AC-B4/rin-B4 groups. Protein processing, in the endoplasmic reticulum, presented in AC-B4/AC-MG and rin-B4/AC-MG. Pyruvate metabolism and phosphatidylinositol signalling system exist in AC-B4/rin-B4 and rin-B4/AC-MG. The above results illustrate that the three pathways play essential roles in the process of phosphorylation regulating tomato fruit.

Protein domain refers to components of similar sequence, structure and function that appear repeatedly in different proteins. Domains typically range in length from 25 to 500 amino acids. The enrichment distribution of DEPs in the classification of protein domains is displayed by bubble chart. In the proteome, the PLAT/LH2 domain is universal in the process of regulating tomato fruit, and especially in the chlorophyll a/b binding protein domain, Cupin 1 and Tubulin were dominant in the process of tomato fruit changing from green to red (Figure S6A–C). In the phosphoproteomic profile, phosphoinositide-specific phospholipase C and EF-hand-like domain were widespread in the process of the phosphorylation regulating tomato fruit (Figure S6D–F).

### 2.8. Enrichment-Based Clustering of DEPs and DEPPs 

Cluster analysis was conducted to find the functional correlation of DEPs and DEPPs in different groups. In the proteome and for molecular functions, the protein expression of sequence-specific DNA binding, glucose-1-phosphate adenylate transferase activity, the structural components of the cytoskeleton, calcium-dependent phospholipid binding, glucosyltransferase activity, glucokinase activity, chlorophyll binding, and oxidoreductase activity were significantly changed in the three different groups (Figure S7A). For cellular components, the protein expression of cell wall, nucleosome, chloroplast thylakoid cavity, photosystem II oxygen evolution complex, and photosystem I reaction center showed remarkably differences in the three different groups, which is consistent with the fruit-ripening phenotype (Figure S7B). For biological processes, the protein expression in photosynthesis, glucan metabolic, cell wall polysaccharide metabolism, carbohydrate biosynthesis, cutin biosynthesis, polysaccharide biosynthesis, energy storage metabolism, and photosynthetic electron transport in photosystem I displayed obvious changes in the three groups, which is agrees with the fruit-ripening phenotype (Figure S7C).

In the phosphoproteome, the protein phosphorylation of chlorophyll binding, pigment binding, sucrose synthase activity, protein histidine kinase activity, phospholipase activity, lipase activity and phosphatidylinositol phospholipase C activity showed an enrichment trend. The processes of carbohydrate transmembrane transporter activity were changed in the three groups (Figure S7D). The enrichment of cellular components found that the protein phosphorylation of cell walls, photosynthetic membranes, chloroplast thylakoid membranes, photosystem II and proteasome complex compounds were obviously different in the three groups (Figure S7E). The enrichment of biological processes found that the protein phosphorylation of the cell protein complex assembly, ethylene biosynthesis, mitochondrial transmembrane transport, organic acid biosynthesis, root system development, the cell response to superoxide and the response to ethylene were remarkably different in the three groups (Figure S7F).

In the proteome, KEGG pathways were mainly enriched in flavonoid biosynthesis, porphyrin and chlorophyll metabolism, the photosynthesis-antennary protein, α-linolenic acid metabolism, unsaturated fatty acid biosynthesis, cutin, amber and wax biosynthesis, glycine, carotenoids biosynthesis, glycolysis, starch and sucrose metabolism, as these were notably different in the different groups (Figure S8A). Protein domains were principally enriched in lipoxygenase, chlorophyll binding protein domain, lipid protein/cytoplasmic fatty acid binding domain, linker histone H1/H5, helix-turn-helix domain, sulfotransferase domain, ribonucleotide reductase-related, hexokinase and ribosomal protein L5 (Figure S8B). In the phosphoproteome, KEGG pathways were mainly enriched in the AGE-RAGE signalling pathway, the MAPK signalling pathway-plant, protein processing in the endoplasmic reticulum, plant hormone signalling and carbon fixation. (Figure S8C). Protein domains were mainly enriched in the cyclic nucleotide binding domain, sucrose phosphatase, the galactose mutant enzyme-like domain, glycoside hydrolase carbohydrate binding, heat shock proteins, ¡sucrose synthase, chlorophyll a/b binding protein domain, ABC transporter type 1, nuclear pore complexes, the NADP-dependent oxidoreductase domain, zinc finger similar to C3HC, enhancer of WD40 repeat sequence, the pyruvate/phosphoenolpyruvate kinase-like domain, the ribosomal protein S5 domain, Hsp90 and phosphatidylinositol-specific phospholipase C. (Figure S8D).

### 2.9. Transcription Factors

**Figure 7:**
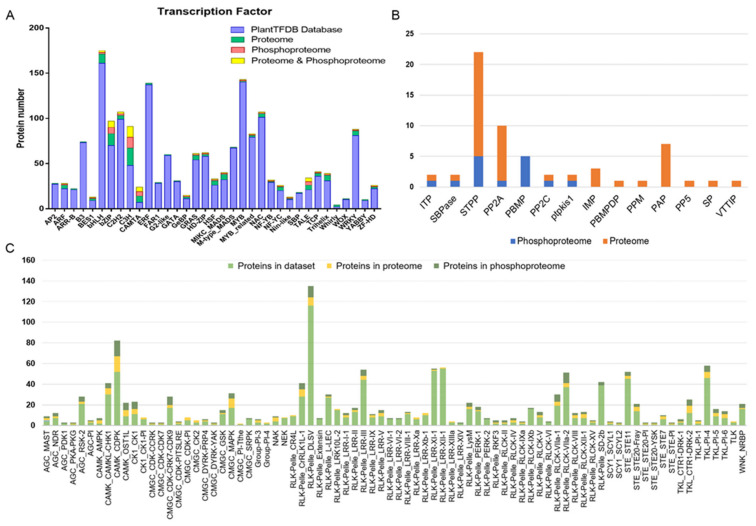
Distribution of transcription factors, phosphatase and protein kinases annotations in the two omics’ profiles and tomato databases. (**A**) Transcription factors, (**B**) phosphatase and (**C**) protein kinases.

Here, 127 transcription factors with significant changes were detected in the proteomic data, belonging to 35 transcription factor families, including C3H, bZIP, bHLH, CAMTA, MADS-BOX, TRIHELIX, GRAS, WRKY, HSF, TALE, C2H2, NAC, TCP, ZF-HD, ERF, HD-ZIP, BES1, MYB-RELATED, WHIRLY, etc. There were three families with more than 10 transcription factors, 19 in the C3H family, 13 in the bZIP family and 10 in the bHLH family. Significantly different proteins, such as IMA, NAC-NOR, MADS-RIN, FSR, ARF2A and so on, belonging to the ZF-HD, NAC, MIKC_MADS, GRAS and ARF families. In addition, AP2, HD-ZIP, B3, MYB_related, bHLH, HSF, CAMTA, Trihelix, WRKY, Whirly and GeBP family proteins were also significantly changeable in the proteome. In the phosphoproteome, the phosphorylation levels of 49 transcription factors were changed, indicating that phosphorylation modification has a widely regulatory effect on transcription activity and the regulation of transcription factors during fruit-ripening. In the phosphoproteome, 49 transcription factors were assorted to 23 different families, containing C3H (12), bZIP (7), CAMTA (5), TALE (4), C2H2(2), ARF, BES1, GeBP, GRAS, HD-ZIP, HSF, MIKC_MADS, MYB, NF-YB, MYB_related, TCP, NF-YC, Nin-like, NAC, Trihelix, WRKY and ZF-HD (1). Among them, the phosphorylation levels of NF-YC, bZIP, HSF, C3H, GeBP, Trihelix, TALE, Nin-like and IMA showed significant difference. In addition, CAMTA, GeBP and C3H showed a higher phosphorylation level than others (Figure 7A) during fruit-ripening. Among them, the phosphorylation levels of fruit-ripening-related protein NOR increased with fruit-ripening. It is worth noting that classic fruit-ripening-related proteins, such as RIN and MC, had expression changes in the proteomic profile, but in the phosphoproteome no changes in phosphorylation levels were detected in this study. The identified phosphorylation sites give useful information for further study of its function at the level of protein modification (Table S8).

### 2.10. Kinases and Phosphatases 

Phosphatases and kinases play important roles in plant development because they can mediate reversible protein phosphorylation. Kinases are regulated by phosphorylation, either by other kinases or autophosphorylation. Tomato protein-kinase databases were provided on the website (iTAK) http://itak.feilab.net/cgi-bin/itak/db_family.cgi (accessed on 30 May 2021). There were 1137 protein kinases in the tomato kinase database, which were divided into 9 groups, AGC, CAMK, CK1, CMGC, PLANT-SPECIFIC, RLK-PELLE, STE, TKL and others. Altogether, 6965 proteins were identified in our proteome, including 182 kinase proteins. A total of 2327 proteins were identified in the phosphoproteome, of which 196 kinases with 493 phosphorylation sites had changes in their phosphorylation levels (8.4%) compared with 2.3% for the whole proteome (as detected), occupying 17.2% of 1137 total annotated kinases, showing that phosphorylation is widely present in kinases during fruit-ripening. Statistical analysis of phosphoproteome kinases showed that AGC-PKA-PKG (1, 100%), CAMK-AMPK (2, 100%), CMGC-CDK-CCRK (1, 100%), CMGC-CDK-CDK7 (1, 100%), CMGC-DYRK-YAK (1, 100%), SCY1-SCYL2 (1, 100%), STE-STE20-Pl (1, 100%), STE-STE20-YSK (1, 100%) and STE-STE-Pl (1, 100%) family represented the largest fraction (100%). Although there were only one or two of these kinases in the databases, they were identified in our proteome and phosphoproteome (Figure 7B), followed by CAMK-OST1L (open stomata-like kinase) (7, 77.78%), RLK-Pelle-RLCK-IV (2, 66.67%), TKL-CTR1-DRK-1 (2, 66.67%) and CK1-CK1 (7, 63.64%). In addition, the phosphorylated proteins in the AGC-MAST, AGC-PDK1, CMGC-CDK-PITSLRE, RLK-Pelle-LRR-XIV, RLK-Pelle-RLCK-IXa, SCY1-SCYL1, TKL-CTR1-DRK-2 and TKL-Pl-1 families demonstrated higher proportion (50%) than other families, suggesting that these kinase families may be prioritized in the phosphorylation regulation of fruit-ripening (Figure 7B).

In addition, 45 phosphatases were identified in our proteomic annotation information, of which 11 phosphatases (with 21 phosphorylation sites) were identified in our phosphoproteome (Figure 7C). Especially, PP2C, SBPase and PTPKIS1 showed the largest proportion (1, 100%), followed by STPP (5, 29.4%) and PP2A (1, 11%).

### 2.11. Prediction of the Phosphorylation Sites of Upstream Kinase and Their Activities 

The higher the level of protein phosphorylation regulated by a phosphokinase, the higher the activity of the phosphokinase. Based on this theory, the regulatory relationship between phosphokinase and phosphorylation sites is used as the gene set, and the phosphate-site expression levels, in the sample, is used as the rank file. The kinase activities of different groups and samples were predicted by GSEA.

**Figure 8:**
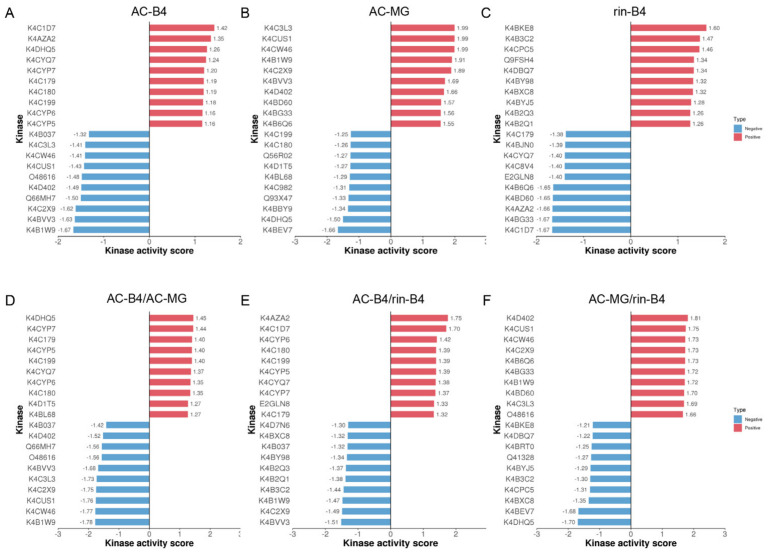
Analysis of phosphokinase activity in different samples and comparison groups. (**A**) AC-B4; (**B**) AC-MG; (**C**) rin-B4; (**D**) AC-B4/AC-MG; (**E**) AC-B4/rin-B4; (**F**) AC-MG/rin-B4. The *x*-axis is the phosphokinase activity score, and the *y*-axis holds the phosphokinases with the top-10 active- or inhibited-state activity scores. Red represents the activated state, and blue represents the inhibited state. The NES (normalized enrichment score) value obtained by the enrichment analysis is as the kinase activity score. If the kinase activity score is more than 1, the kinase tends to be activated; If the kinase activity score is less than 1, the kinase tends to be inhibited. The ratio threshold of the differential expression change ratio is 1.5, and the difference significance *p* value threshold is 0.05.

Figure S10 shows that there were more kinases in the activated state than in the inhibited state in the AC-MG and rin-B4 samples, while there were more kinases in the inhibited state than in the activated state in the AC-B4 samples. In the AC-B4/AC-MG and AC-B4/rin-B4 groups, the number of kinases in their inhibited state was remarkably greater than of those in their activated states, while in the rin-B4/AC-MG group, the activated-state kinases were significantly more common than inhibited-state kinases. In the different samples (Figure 8A–C), the kinase activities of K4C1D7, K4AZA2 and K4CYQ7 were suppressed in AC-B4 and rin-B4. K4DHQ5, K4C3L3, K4CW46, K4CUS1, K4D402, K4C2 × 9, K4BVV3 and K4B1W9 were significantly different in AC-B4 and AC-MG and were activated. K4BD60, K4BG33 and K4B6Q6 were activated in AC-MG, and, in rin-B4, were in their inhibited states.

In different groups (Figure 8D–F), K4DHQ5, K4D402, K4C3L3, K4CUS1 and K4CW46 were suppressed in AC-B4/AC-MG and AC-MG/rin-B4. K4CYP7, K4C179, K4CYP5, K4C199, K4CYQ7, K4CYP6; K4C180 in AC-B4/AC-MG and AC-B4/rin-B4 were all activated; and K4B037 and K4BVV3 were all inhibited. The kinases K4C2X9 and K4B1W9, in the three groups, were inhibited in AC-B4/AC-MG and AC-B4/rin-B4 but activated in AC-MG/rin-B4. The kinase activity cluster heat map showed the commonality of kinase activity in the same phenotype, or the specificity in different phenotypes (Figure S9). Explore the potential connection between kinase activity and phenotype, and screen interested kinases will provide reference for subsequent experiments. K4B037, K4C3L3, K4CW46, K4CUS1, O48616, K4C2X9, K4BVV3, K4B1W9, K4CYP7, K4C179, K4C180, K4C199, K4CYP6 and K4CYP5 were all remarkably enriched in different samples and the active kinase group.

### 2.12. The Relationship between Kinases and Substrates

According to the regulatory relationship between kinases and phosphorylation sites, for each group, phosphokinases with significantly activated or inhibited activity and phosphorylation sites with significantly different expression levels were screened to construct a kinase regulatory network, which can visually observe kinases and substrates. Based on the complex regulatory relationship between phosphokinase and substrate, a kinase regulatory network was constructed and visualized. In the AC-B4/AC-MG group (Figure S11), K4BZ85 is an inhibitory kinase. The corresponding substrates included 19 downregulated proteins and five upregulated proteins. The upregulated site protein K4DAP9-166T and the downregulated site protein K4CV09-97S were also substrates of many kinases. K4DAX8-427S corresponded to seven kinases in activated states. Down-regulation sites protein Q93X44-353S, K4BJZ4-310S and K4D4F9-214S were substrates of 34 inhibitory kinases; In the AC-B4/rin-B4 group (Figure S12), K4BZ85 kinase corresponds to seven up-regulated proteins and 15 down-regulated proteins. K4B0M2-266S was the substrate of 16 inhibited kinases, K4DAP9-166T was the substrate of seven activated kinases. In the AC-MG/rin-B4 group (Figure S13), K4BZ85 corresponded to five down-regulated proteins and eight upregulated proteins. The 13 kinases in the activated state were all found to regulate K4CV09-97S. The 10 inhibitory kinases correspondingly regulated K4B3M6-16T as its substrates. K4CBQ3-70S, K4CM5-231S and K4BZ85-71S all corresponded to three different kinase substrates. These results show that the relationship between kinase and substrate is not one-to-one, and the above-mentioned kinases and substrates may play pivotal roles in fruit-ripening

### 2.13. Plant Hormone Signal Transduction in the Proteome and Phosphoproteome

**Figure 9:**
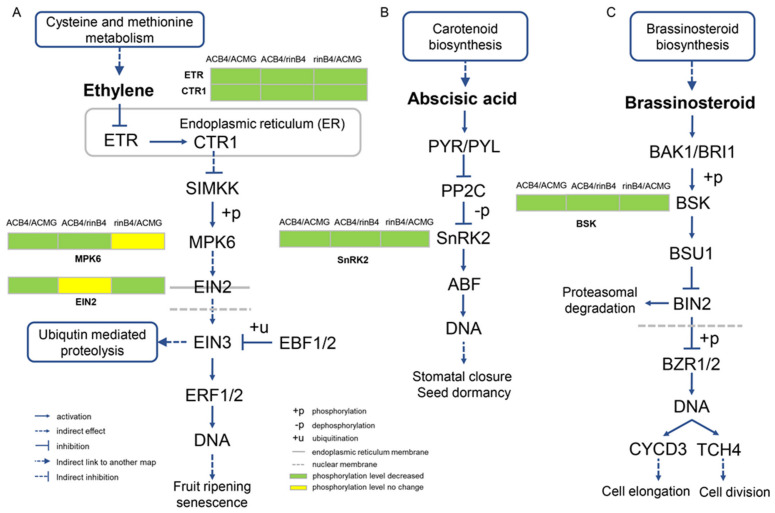
Comparative analysis of ethylene, ABA and the BR signalling-transduction pathway phosphoproteins from different comparison groups. (**A**) Ethylene, (**B**) ABA and (**C**) BR signalling-transduction pathway. MG, mature green; B4, breaker + 4. ETR, ethylene resistant; CTR1, constitutive triple response 1; SIMKK, mitogen-activated protein kinase 4/5; MPK6, mitogen-activated protein kinase 6; EIN2, ethylene insensitive 2; EIN3, ethylene insensitive 3; EBF1/2, EIN3-binding F-box 1/2; ERF1/2, ethylene-responsive factors 1/2; PP2C, protein phosphatase 2C; SnRK2, SNF1-related protein kinase 2; ABF, ABA responsive element binding factor; BAK1, brassinosteroid insensitive 1-associated receptor kinase 1; BRI1, protein brassinosteroid insensitive 1; BSK, BR signalling kinase; BSU1, serine/threonine-protein phosphatase; BIN2, protein brassinosteroid insensitive 2; BZR1/2, brassinosteroid-resistant 1/2; CYCD3, cyclin D3; TCH4, xyloglucan: xyloglucosyl transferase TCH4.

Most plant hormones are regulated by phosphorylation during signal transduction (Figure 9). In the proteome, in AC-B4/AC-MG group, the protein expression of oxidative phosphorylation, photosynthesis, antenna protein, carbon fixation process and eukaryotic ribosome biogenesis were reduced, while protein expression in pathways such as fatty acid metabolism, sugar and starch synthesis, linolenic acid metabolism, carotenoid synthesis, ABA synthesis, flavonoid biosynthesis and unsaturated fatty acid synthesis was increased. In the AC-B4/rin-B4 group, protein expression of photosynthesis and antenna protein were decreased, while protein expression in fatty acid synthesis, steroid synthesis, cysteine methionine metabolism, amino acid synthesis, cyano amino acid (ethylene precursor) synthesis, flavonoid synthesis, carotenoid synthesis and the unsaturated fatty acid pathway was increased. In the rin-B4/AC-MG group, protein expression in photosynthesis and the antenna protein and ribosomal RNA assembly processes were decreased, while protein expression in linolenic acid metabolism, carotenoid synthesis and ABA synthesis pathway was increased. The changes in ethylene and ABA in the MAPK pathways was not significant.

In phosphoproteome, AC-B4/AC-MG group, the MAPK pathway, the phosphorylation level of CTR1, MPK3/6 and EIN2 protein in the ethylene signalling pathway, and the MPK6 protein in the Jasmonic acid signalling pathway were decreased, ultimately affecting the stress resistance of plants. In plant hormones signal transduction, phosphorylation of SnRK2 (ABA signalling pathway) and BSK (Brassinolide pathway) was also decreased. In the AC-B4/rin-B4 group, in the MAPK pathway, the phosphorylation levels of ETR1/ERS, CTR1, MPK3/6 proteins in the ethylene signalling pathway, the MPK6 protein in the Jasmonic acid signalling pathway and SnRK2 and MPK6 of the ABA signalling pathway were decreased. The phosphorylation level of SnRK2 in the ABA signalling pathway was reduced, which affects stomata and seeds. In ethylene signalling, the phosphorylation of ETR, as well as of CTR1 and MPK6, was reduced, which affected fruit-ripening and senescence. The phosphorylation of BSK in the brassinolide pathway was decreased, which affected cell elongation and differentiation. Phosphorylation of the age signalling pathway was also decreased. In the rin-B4/AC-MG group, MAPK signal pathway, the phosphorylation levels of ETR1/ERS and the CTR1 protein in the ethylene signal-transduction pathway were increased, and EIN2 was decreased, which ultimately affected the stress resistance of plants. In the ABA signal pathway, the phosphorylation level of SnRK2 was decreased. Age signalling-pathway protein phosphorylation levels and endoplasmic reticulum protein-processing levels were decreased.

### 2.14. Protein and Phosphorylation Changes in Other Signalling Pathways

**Figure 10:**
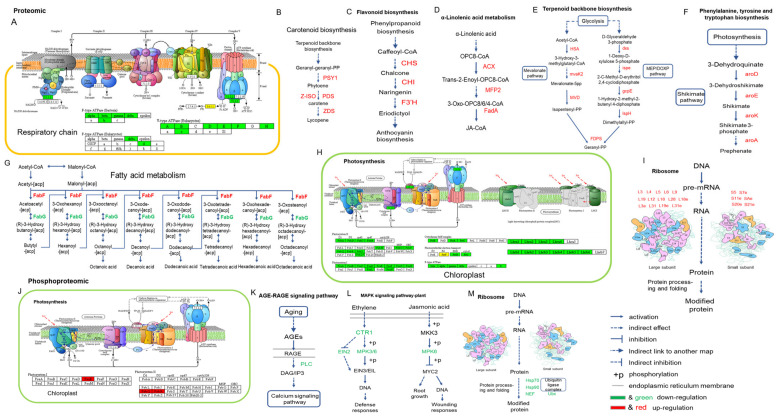
Schematic diagram of representative DEPs and DEPPs in metabolic pathways during fruit-ripening. (**A**) Respiratory chain, (**B**) Carotenoid biosynthesis, (**C**) Flavonoid biosynthesis, (**D**) α-linolenic acid metabolism, (**E**) Terpenoid synthesis, (**F**) Phenylalanine, tyrosine and tryptophan biosynthesis, (**G**) Fatty acid metabolism, (**H**) Photosynthesis, (**I**) Ribosome assembly, (**J**) Photosynthesis (Phosphoproteomic), (**K**) AGE-RAGE signaling pathway, (**L**) MAPK signaling pathway, (**M**) Ribosome assembly (Phosphoproteomic). The metabolic pathways are constructed from on proteomics and phosphoproteomics data. The red fonts represent up-regulation, green fonts represent down-regulation.

Enrichment of the proteome KEGG found that, compared with un-ripening fruits, the expression of key enzyme proteins in photosynthesis, the respiratory chain and fatty acid metabolism were all down-regulated in ripening fruits, while the main proteins in the carotenoid synthesis pathway, flavonoid synthesis pathway, linolenic acid metabolism process, terpenoid synthesis, amino acid synthesis and ribosome assembly process were all up-regulated (Figure 10). The carotenoid synthesis pathway, flavonoid synthesis pathway, linolenic acid metabolism process, terpenoid synthesis, shikimic acid pathway, amino acid synthesis, and ribosome assembly process were enhanced in fruit-ripening, which agreed with the appearance of colour in the fruit. In the phosphoproteome, compared with unripening fruits, the phosphorylation of important proteins in photosynthesis were increased after fruit-ripening, which is negatively correlated with protein expression. The phosphorylation of proteins in the age signalling pathway, plant MAPK signalling pathway and in protein processing were all decreased, indicating that the phosphorylation levels of most pathway proteins was down-regulated during ripening. This has not yet been reported.

### 2.15. Protein-Protein Interaction Network Related to Fruit-Ripening

**Figure 11:**
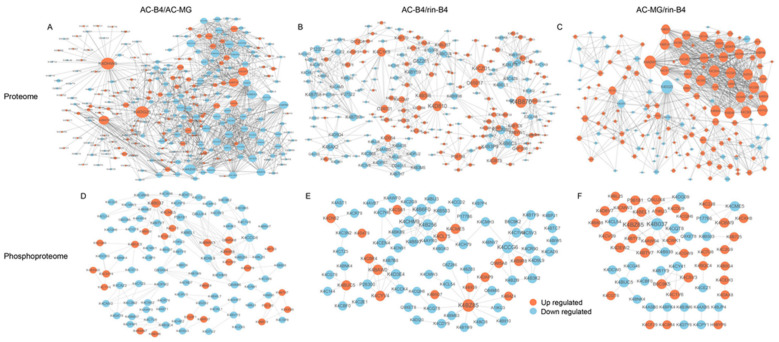
Protein–protein interaction network. Protein–protein interaction network for proteins corresponding to representative DEPs (proteome, [**A**–**C**]) and DEPPs (phosphoproteome, [**D**–**F**]) in different comparison groups.

In the proteome, in AC-B4/AC-MG (Figure 11A), up-regulated protein K4DHW5, K4DG25 and K4B870 corresponded to the most-interacting proteins, followed by K4D3F8, K4BP30; the down-regulated proteins K4ASW1, K4BEK3 and K4C3E8 corresponded to the most-interacting proteins, followed by K4DEP5, K4AYG, K4WB0 K4B6C3 and K4CHR6. In AC-B4/rin-B4 (Figure 11B), the up-regulated proteins K4B870, K4D810, K4BSI6, K4C1K9, O65917, K4CZD1 and O24031 corresponded to the most-interacting proteins, followed by K4D054, K4DBT9 and P08196; the down-regulated proteins K4BY59, G8Z261, K4B3P9, K4B6C3, K4BAW0, K4B7S8 and K4BAX2 corresponded to the most-interacting proteins, followed by P12372, K4BEL1 and K4BLR5. For AC-MG/rin-B4, in Figure 11C, the upper-right corner of the interaction graph shows that there many correspondently up-regulated proteins; K4ASW1, K4CAE2, K4CSH4, K4CQE5, K4BB40, K4C3E8 and K4AYG3 corresponded to the most-interacting proteins, followed by K4CVS3 and K4DHU7; the down-regulated protein K4DG25 corresponded to the most-interacting proteins, followed by K4D3F8, and so on. It is worth noting that K4DG25, K4B870, K4BPB0 and K4ASW1 had many corresponding proteins in two or more groups, indicating that the regulation of these proteins corresponds to a crucial link in fruit-ripening regulation.

The phosphoproteome has a much smaller number of differential phosphoproteins. AC-B4/AC-MG (Figure 11D) had the most up-regulated protein, K4B037, which corresponded to the most-interacting protein, followed by K4CME5, K4BMW3, K4B9R8, Q9M5A8 and K4BMN7. There is a little difference in the number of interacting proteins corresponding to down-regulated proteins. K4D9L9, K4CCG6, P36181 and K4BTV7 corresponded to the most-interacting proteins. In particular, the number of down-regulated phosphorylation proteins was greater than the number of up-regulated phosphorylation proteins. In AC-B4/rin-B4 (Figure 11E), the up-regulated protein K4BZ85 was the most-interacting protein, followed by K4CYV4 and K4BAW0. The down-regulated proteins K4CHM9, K4B256, K4B6F0 and K4CCG6 corresponded to the most-interacting proteins, and the number of interacting proteins corresponding to other proteins was not significantly different. In this group, there were more phosphorylation-level down-regulated proteins than up-regulated proteins. In AC-MG/rin-B4 (Figure 11F), K4BZ85 corresponded to the most-interacting proteins, followed by K4DEW2, K4BTV7, K4CV09 and K4C8W9. The down-regulated protein K4B037 corresponding to the most-interacting protein, followed by K4BUC5, K4B037, K4CME5, K4BAW0, K4BZ85 and K4CCG6 (Table S9). There were a lot of corresponding proteins in two or more groups, illustrating that the changes of phosphorylation of these proteins corresponds to important links in fruit-ripening regulation.

## 3. Discussion

Fruit-ripening is a sophisticated process. However, the regulatory mechanism of ethylene-insensitive climacteric fruits remains unclear. In this study, through phosphoproteomics and high-throughput proteomics, some of the complex regulatory of AC^++^ (wild-type) and *rin* mutant fruit ripening were revealed.

### 3.1. Two Omics’ Analyses Add New Post-Translational Data for Tomato Fruit-Ripening

A total of 6965 and 2327 proteins were certified in the proteome and phosphoproteome, separately. The amount of protein obtained, here, is the largest throughput of the tomato proteome reported so far. By the two omics’ analyses, we have identified 7856 proteins, in total, from tomato fruit. By comparing the phosphopeptides identified from the three different stages of tomato fruit and their corresponding phosphoproteins, 2327 phosphopeptides and 1853 phosphoproteins were determined across all three different fruit stages, showing that the majority of phosphopeptides and phosphoproteins appear at all phases of tomato ripening. In the Plant Protein Phosphorylation Database (P3DB) [33] *Arabidopsis thaliana*, *Solanum tuberosum*, *Oryza sativa*, *Zea mays*, *Medicago truncatula*, *Vitis vinifera*, *Glycine max*, *Brassica napus* and *Nicotiana tabacum* data have been included, and there is no relevant data for tomatoes. Therefore, our data has not only significantly expanded the phosphorylation database of plant proteins, but has also provided a new reference for the functional study of phosphorylated proteins during tomato fruit-ripening.

### 3.2. Phosphorylation Play an Important Role during Fruit-Ripening

The main amino acids that affect phosphorylated protein function are tyrosine, threonine and serine [34]. Of the 4842 non-redundant phosphorylation sites, the amount of phosphotyrosine (pTyr), phosphothreonine (pThr) and phosphoserine (pSer) were 233 (5%), 592 (12%), and 4017 (83%), respectively (Figure 1E). Phosphoproteome analyses of wheat [35], maize [36], *Arabidopsis* [37] and Pepper [38] have shown that Tyr phosphorylation sites (less than 2%) and Ser phosphorylation sites (more than 80%) have similar distribution. The phosphorylation site of Tyr was 5% in our omics analyses. Even though Tyr phosphorylation had a low occupancy rate, it still played important roles in the examined plants. For example, a phosphoproteomic study of *Arabidopsis thaliana* pollen detected a pTyr-containing peptide [39]. In the present work, 197 proteins with 233 pTyr sites were found in the phosphoproteome, in which 35 proteins contained more than one pTyr site. In addition, the Tyr of SlMEKK1, SlSNRK2, SlEIN2 and SlMPK1 proteins in the MAPK pathway were phosphorylated. These proteins, Tyr phosphorylated, have been reported in rice. SlMEKK1 and SlSNRK2 were previously only reported to be Ser/Thr protein kinases, yet their Tyr was also phosphorylated in our case. Moreover, there were five transcription factors in these Tyr-phosphorylated proteins. Thus, these results reveal that Tyr phosphorylation may play a vital role in the MAPK signal cascade that regulates fruit-ripening.

Regarding phosphorylation sites, although most proteins (1306 proteins) only have one phosphorylation site, many proteins with more than 15 phosphorylation sites have been identified (Figure 1F). Solyc11g072340.2.1, whose function is unidentified, has the largest number of phosphorylation sites (22). It is located in the nucleus and encodes a protein containing an RNA recognition-motif domain, which may be related to RNA variable splicing. Solyc02g031860.3.1, of unidentified function, contains 16 phosphorylation sites, is located in the nucleus and encodes a protein containing the PB1 domain. It is predicted to have tyrosine kinase activity. These results were consistent with rice anthers [40]. Solyc11g006000.2.1 contains nine phosphorylation sites, encodes MAP3K, a protein containing a protein-kinase domain. Solyc08g074240.3.1 contains eight phosphorylation sites, which encodes 40S ribosomal protein S6. In particular, ACO3, which is located in the nucleus, contains eight phosphorylation sites and encodes 1-aminocyclopropane-1-carboxylate oxidase, a vital step in ethylene synthesis. The above results indicate that important proteins related to biological processes, such as alternative splicing, kinase, ribosomal protein and ethylene synthesis, can regulate tomato fruit-ripening through phosphorylation.

Figure 1G showed the profile of the number of phosphorylation sites in the phosphorylated proteins; 56% of the phosphorylated proteins contained one phosphorylation site, and 22% of the phosphorylated proteins contained two phosphorylation sites. About 4% (90 phosphorylated proteins) had seven or more phosphorylation sites. Of all the identified phosphorylated proteins, 49 transcription factors were included (Table S1), indicating that protein phosphorylation is extensively present in transcription-regulation networks.

### 3.3. Motif and Subcellular Locations of Phosphoproteins in Tomato Fruit

There were 50 phosphorylation motifs defined in our data, including 38 pSer motifs, eight pThr and four pTyr motifs (Figure S3A, Table S2). Liu et al. [38] defined 27 phosphorylation motifs, involving five pThr motifs and 22 pSer motifs, in the phosphoproteome of pepper fruit development, but did not define the pTyr motif. In the phosphoproteome of mutant cotton fibre development, two pThr and 17 pSer were defined, while pTyr was also undefined [41]. In our data, [RxxxY] and [SxxxxY] were found in the highest proportions, with 41 and 37 peptides, respectively, in four pTyr motifs. This showed that pTyr plays a remarkable role in tomato fruit-ripening. Among the 38 pSer motifs, [SP], [GS] and [RxxS] motifs were found in the highest proportions, with 342, 227 and 207 peptides, respectively. Many studies have reported that [SP] and [Rxxs] are frequently repeated motifs [42,43]. In the plant stress response, SnRK2.6 recognizes the GS motif [44]. [SP] motifs are the possible substrates of cyclin-dependent kinases, CDK-like kinases and MAPK, and calcium/calmodulin-dependent PK II [45] can recognize [Rxxs]. In these motifs, [RxxSxSP] has the lowest proportion of phosphorylated peptides, accounting for only 20 of confirmed peptides. Among the eight pThr, [PxTP] and [TP] accounted for the highest proportions of 78 and 97 peptides, respectively. Motifs [RxxTP] and [LxRxxT] appeared in the lowest proportions, at 24 identified phosphorylated peptides (Figure S3 and Table S5). It is possible that the threonine/serine surrounding the alkaline and neutral residues (R and P) are more easily phosphorylated in tomato fruits.

For subcellular location, previous studies have shown that all subcellular regions contain protein phosphorylation and more than 40% of protein phosphorylation occurs in the nucleus [38,46], which is consistent with our findings. Proteins were mostly localized in the nucleus and cytoplasm, in our research, which may be due to the localization of the proteins including an [SP] motif in the nucleus and cytoplasm [43]. In rice [47], in addition to the nucleus, the chloroplast is the most localizing organelle. We conjecture that different parts of the organ in the sample may have caused this difference. The samples here-studied were fruits; compared with leaves, fruits have fewer chloroplasts, and fewer chloroplast functions in the later stages of fruit development, resulting in lesser chloroplast protein localization.

### 3.4. Comprehensive Analysis of DEPs and DEPPs during Tomato Fruit-Ripening

Tomato fruit colour varies most from MG to B4. Therefore, the expression of proteins and phosphoproteins also changed greatly between MG and B4 fruits. At different phases, DEPs and DEPPs in AC-B4/AC-MG, primarily, are regulated, revealing that, after the MG phase, the expression of certain proteins and phosphoproteins showed obvious changes in the fruit, thereby promoting its ripening. In AC-B4/rin-B4, the number of the DEPs, as well as the number of the DEPPs, was less than that in AC-B4/AC-MG, which indicates that, due to the mutation of RIN in *rin*, the expression of some proteins participating in tomato ripening are affected. That rin-B4/AC-MG had the least DEPs indicates that, due to the mutation of RIN, the rin-B4 and normal MG fruits were not significantly different, which further indicates that the mutation of RIN strongly inhibits tomato fruit-ripening. The distribution of proteins between the two omics shows that the phosphorylation of tomato fruits plays a crucial part in the ripening process from MG to B4, and that *rin* inhibits fruit-ripening. It is worth noting that rin-B4/AC-MG had more DEPPs than other groups, indicating that RIN may be closely related to phosphorylation regulation during fruit-ripening.

In the proteome, ACO3, LeEXP1, GSH1 and LeSRK2C were significantly increased in all groups. ABA signal transduction pathway is intensely associated with the SNF1-related kinase [48]. The pivotal enzyme in the ethylene synthesis pathway is ACO3 [49]. LeEXP1 is an important fruit cell wall-development protein [50]. These results indicate that changes in ethylene biosynthesis, cell-wall development, the ABA signalling pathway and glutamate–cysteine ligase may play crucial regulatory components during tomato fruit-ripening. In the phosphoproteomic profile, 40S ribosomal protein S6 (K4CME5), auxin repressed/dormancy associated protein (Q0PY39) and ACO3 were significantly increased in all groups. The 40S ribosomal protein S6 is an important part of the small ribosomal subunit. The mRNA binding site of the cytoplasmic ribosomal 40S subunit is the location of RPS6 [51]. These results suggest that the assembly of ribosomes, ethylene synthesis and auxin response play a key part in tomato fruit-ripening.

Further analysis of the protein list of the GO classification, in the proteome, in AC-B4/AC-MG and AC-B4/rin-B4, E4, Ca3, cell wall-related proteins PG2, Cel2, LeEXP1, MAN4, ethylene synthesis related ACO1, ACO3, ACO4, ACO5, the synthesis of linoleic acid flavour substance LOX1.2, the related enzymes of pigment synthesis CHS2, CRTISO, PSY1 and ZDS, cellular ribonuclease RNALX and phosphokinase LeSRK2C, LeCDPK2 were all significantly changed during fruit-ripening. In the phosphoproteome, PSBR, K4CLP5, 40S ribosomal protein S6, ACO3, LeGAD2, serine/threonine-protein phosphatase, PsbQ, psbH, phosphoglycerate kinase, sucrose synthase, LHA1, S-acyltransferase, LeCDPK2, CYP, MRLK1, LET12, MPK1, phosphoinositide phospholipase C, ACO1, LeSRK2C, LeCCH, CDKC, PURA, Chlorophyll a-b binding protein and ETR4 were all significantly changed. These results indicate that, during fruit-ripening, cell-wall metabolism, ethylene synthesis, flavour-compound synthesis, pigment synthesis, ribonucleic acid and phosphokinase all play important roles. These proteins play vital roles in cell-differentiation, cell-development and cell-cycle regulation. Accordingly, the high expression of these phosphoproteins results in accelerated cell development and differentiation, shortened cell cycles and benefits to fruit expansion and growth. As fruits continued to grow, the abundance of these phosphoproteins decreased, cell development and differentiation slowed and the fruits gradually ripened.

Moreover, in the proteome, CAB1B (LHCB1), LHCB4, LHCB5, LHCA1, LHCA2 (CAB7), HCA3(CAB8), LHCA4, psbB, psaC, PsbQ, PSBR and psaA were noteworthy decreased in all groups. In the phosphoproteome, LeSRK2C, cysteine proteinase inhibitor (4BJE4), phosphoinositide phospholipase C (K4C1V6 and K4C5V3), LHCB1, protein kinase MRLK1 (K4CS51) were significantly decreased in all groups. These proteins are closely related to ABA signal transduction, chlorophyll metabolism or photosynthesis, indicating that these pathways play vital roles in tomato fruit-ripening. The above results suggest that the most active processes of protein and phosphorylation regulation show consistent trends during fruit-ripening, and they were mainly concentrated in cell metabolism. Considering that most of the key molecules during fruit-ripening are related to active cell metabolism, these proteins are ideal candidates for studying the mechanisms controlling fruit-ripening at the protein- and post-translational-modification levels.

As seen from Figure 4, except for the ‘POORLY CHARACTERIZED’ (function unknown and general function-prediction only), the functions of differential proteins in the proteomic profile mainly were intracellular trafficking, chaperones, post-translational modification, translation, signal transduction mechanisms and biogenesis and ribosomal structure. In the phosphoproteomic, the classification of proteins corresponding to phosphorylation differences was also mainly concentrated in post-translational modification and RNA processing of these functional clusters. These results indicate that signal transduction mechanisms and post-translational modification play vital regulatory roles in tomato fruit-ripening.

KEGG pathway enrichment showed that, during normal fruit-ripening, genes encoding cutin and cell walls, photosynthesis, oxidative phosphorylation, plant ribosome generation, phagosome-related proteins, and ABA synthesis were mostly down-regulated. Fatty acid synthesis and degradation, amino acids syntheses, glutathione metabolism, starch and sugar metabolisms, terpenoid skeleton synthesis, carotenoid synthesis, flavonoid synthesis, linolenic acid synthesis and unsaturated fatty acid synthesis, were all up-regulated, further indicating that these pathways play essential roles during tomato fruit-ripening. In the same period, between the *rin* fruit and the normal-ripening fruit, in addition to the changed trends of the above pathways, ethylene synthesis, sulfur metabolism, glycolysis pathway, lysine synthesis and glucosinolate synthesis, etc. all showed an up-regulatory trend, indicating that these genes and pathways may be regulated by RIN.

Since chlorophyll degradation, carotenoid synthesis and cell-wall metabolism are important processes for fruit-ripening, the identified changes in their phosphorylation levels can accelerate the study of the molecular mechanisms of post-translational regulation. Ethylene signal transduction and MAPK signal transduction were mainly involved in signal transduction, which is mainly focused on post-translational control. These phosphorylated proteins and phosphorylation sites will help to reveal the functions and regulations of signal transduction at level of protein modification.

### 3.5. Transcription Factors and Their Phosphorylation during Fruit-Ripening

In this study, during tomato fruit-ripening process, 127 transcription factors with significant changes were detected. In the proteome, they belonged to 35 transcription-factor families, including C3H, bZIP, bHLH, CAMTA, MADS-BOX, TRIHELIX, GRAS, WRKY, HSF, TALE, C2H2, NAC, TCP, ZF-HD, ERF, HD-ZIP, BES1, MYB-RELATED and WHIRLY. Among these families, there were three families with more than 10 transcription factors; the C3H (19), bZIP (13) and bHLH (10) families. The above results indicate that, during tomato fruit-ripening, these transcription factors can not only regulate gene transcription at the beginning of transcription, but also play a significant role in protein levels after transcription. NAC-NOR and MADS-RIN played essential roles in the fruit-ripening process, thus validating prior research [5]. In the phosphoproteomic, phosphorylation levels of 49 protein were changed, suggesting that protein phosphorylation is extensively present in the regulation and modification of TFs during fruit-ripening, potentially regulate their transcriptional activity. The phosphorylation level of NAC-NOR increased with fruit-ripening, while classic proteins related to fruit-ripening, such as RIN and MC, were only expressed in the proteome data. The 115 transcription factors identified in the phosphoproteome are classified into 32 different families, including C3H (21), bZIP (12), C2H2(10), MYB_related(7), CAMTA(5), bHLH, HB-other, HSF, NAC, TALE and WRKY (4). Interestingly, the phosphorylation ratios of the C3H, CAMTA, GeBP, Nin-like, ARF and trihelix subfamilies were higher than of other subfamilies, indicating that these subfamilies might play more vital roles in fruit-ripening.

### 3.6. Phosphorylation of Phytohormone Signal Transduction and MAPK Signal Pathway in Tomato Fruit-Ripening

Protein phosphorylation and dephosphorylation play key roles in ethylene signal transduction [52]. The phosphorylation levels of ETR and CTR1 did not change significantly during tomato fruit development (Figure 9), suggesting that ethylene does not affect the phosphorylation of these two proteins during tomato fruit development. It is speculated that CTR1 can activate ethylene signals through the MAPK cascade [53,54]. EIN2 was a positive regulator of the ethylene response, [55] whose functional defect mutants exhibit an ethylene-insensitive phenotype in *Arabidopsis thaliana* [56]. ERFs are transcription factors; EIN3 could bind to PERE sites to induce gene expression. PERE sites are cis-acting factors of the ERF1 promoter. ERF1 can bind to GCCbox of many secondary ethylene responsive gene promoters to induce gene expression and cause the ethylene response [57]. When ethylene concentration in the cell is insufficient, ETR activates CTR1; then, the activated CTR1 phosphorylates the C-terminus of EIN2 and inactivated it [58], restraining the downstream ethylene signal and reaction. Consequently, at the MG stage, when the ethylene content was low, the phosphorylation of EIN2 and its downstream ERF1 and upstream MPK6 were higher. However, high concentrations of ethylene in the cell caused the receptor to bind to the hormone and inactivate it, which in turn turned off the CTR1 protein. The phosphorylation of the positive regulatory protein, EIN2, can be blocked by a series of phosphorylation cascades by the inactivated CTR1-encoded protein. Consequently, the phosphorylation of EIN2 was decreased. Chen et al. [59] proved that, at multiple threonine and serine residues, EIN2 is not phosphorylated in the presence of ethylene.

By an unknown mechanism, the C-terminal of EIN2 divides and migrates to the nucleus, and EIN3/EIL1 is stabilized, inducing degradation of ERF1/2, EIN3 and EIN2 cause an ethylene response [60]. Consequently, when ethylene content increased at stages of B and B4, the phosphorylation levels of MPK6 and EIN2 proteins in the signal chain were significantly lower at stage B than at stage MG, even though there were two, six and four phosphorylation sites, respectively. This indicates that ethylene signal transduction may involve a phosphorylation cascade during tomato fruit development. Meanwhile, it was found that the phosphorylation level of EIN3 regulated the ethylene response and did not significantly change over the entire process of fruit development. This suggests that ethylene signalling may be mainly affected by the phosphorylation levels changes in MPK6 and EIN2.

### 3.7. Protein Kinases and Phosphatases in Tomato Fruit-Ripening

Reversible protein phosphorylation plays a vital role in plant development [61]. Kinases are generally regulated by autophosphorylation or phosphorylation of other kinases [62]. In this study, protein kinases were widely identified in the phosphoproteomic (accounting for 8.4% of the total recognition proteins), indicating that these kinases may play essential roles in the phosphorylation regulation of tomato fruit ripening. In our phosphoproteomics data set, a total of 196 kinases were identified with 3615 phosphorylation sites, accounting for 21.88% of the 896 annotated kinases in the tomato protein kinase database. This is the largest percentage of kinases and phosphorylation sites ever identified, for instance, compared with rice [40]. To determine the phosphorylation regulatory networks of the kinase family, enrichment analysis was performed. Compared with the total number of recognized kinases, the RLK family had the largest proportion (84.42%), followed by CAMK (29.14.8%), CMGC and TKL (22, 11.2%). In addition, the proportion of phosphorylated proteins in the AGC and STE families was higher than that in other families, suggesting these kinase families might be prioritized in the regulation of phosphorylation in tomato ripening. Additionally, 11 phosphatases were identified with 21 phosphorylation sites, of which STPP phosphatases accounted for the largest proportion (5, 45.5%), followed by PP2A, PP2C and PBMP (1, 9%).

Receptor-like kinases, which widely control the process of plant development, are a vital part of plant cells’ signal transduction. Nine RLKs, including SERK1, BAM2, SERK2, EMS1, ERL2, ER, ERL1, RPK2 and BAM1, play key roles in the development of *Arabidopsis* anthers [63]. Of tomato fruits’ phosphorylated proteins, 84 RLKs of 30 subfamilies were identified and 165 phosphorylation sites were detected. The percentage of phosphorylated proteins in the LRR and RLCK subfamilies (7.1% and 5.6%, respectively) was much higher than in the other subfamilies, suggesting that these proteins may be necessary for fruit development and are regulated by phosphorylation. CTR1-like protein kinase 4 affects fruit development by regulating ethylene synthesis and response [64]. Receptor-like kinase mutations affect male sterility and induce parthenocarpy in tomato [65]. The ABA receptor FaABAR interacts with the protein kinase FaRIPK1 to affect the fruit-ripening of the strawberry [66]. SNF1 and trehalose 6-phosphate-associated protein kinase 1 inhibit the formation of the cucumber primary fruit [67]. The above results and related studies provide important evidence for kinases’ regulation of fruit development and ripening through phosphorylation. Phosphatase plays the role of dephosphorylation, maintaining and balancing the activity of phosphorylation regulation together with kinases. Yet, there are few studies have sought to prove the function of phosphatases during fruit development and ripening. Among the 45 identified phosphatases, there were eight phosphatases with at least three phosphorylation sites, which directly proves that phosphorylation regulates phosphatase activity during fruit-ripening.

**Figure 12:**
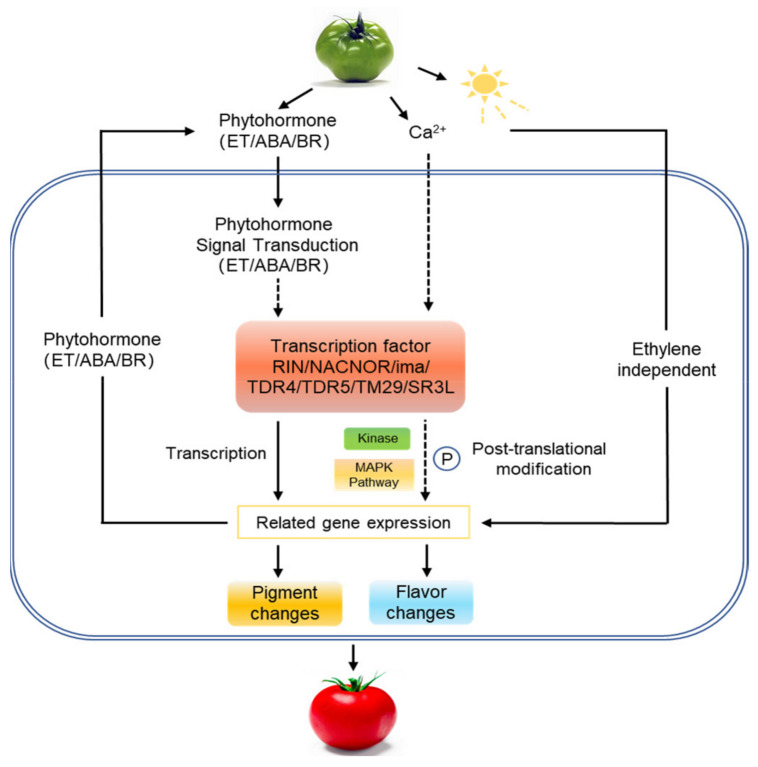
An overview of the fruit-ripening network examined in this study. The ripening of fruit is a process of the co-regulation of plant hormones, light, Ca^2+^ and other factors. These factors can influence the regulation of the expression of transcription factors of related functional genes through phosphorylation modification, influencing the activity of fruit-ripening-related proteins, and thereby regulate the fruit-ripening process.

In summary, combining high-throughput proteomics and phosphoproteomics (Figure 12), we have shown that ethylene biosynthesis and signal transduction, photosynthesis regulation, carotenoid and flavonoid biosynthesis, chlorophyll degradation, and ribosomal subunit expression changes, MAPK pathway changes and transcription factors, as well as kinases, all play essential roles in tomato fruit-ripening and are regulated by phosphorylation. The affected protein levels are correlated with the corresponding gene transcript levels, such as NAC-NOR, MADS-RIN, IMA, TAGL1, MADS-MC and TDR4. The modulated accumulation levels of these proteins agreed with phenotype during fruit-ripening. The phosphorylation of NAC-NOR and IMA is involved in the regulation of tomato fruit ripening. A variety of primary and secondary metabolic pathways, such as glycolysis, fatty acid metabolism, vitamin metabolism, and isoprenoid biosynthesis, are involved in the regulation of fruit ripening, although photosynthesis is inhibited in this process. In particular, the discovery and analysis of kinases and their regulatory networks during fruit-ripening will further provide important data for the research on kinases related to fruit-ripening. These data constitute a map of protein and phosphorylation in the regulation of tomato fruit-ripening, which will lay the foundation for in-depth studies of the sophisticated molecular mechanisms of fruit-ripening and provide guidance for molecular breeding.

## 4. Materials and Methods

### 4.1. Growth Conditions and Plant Materials 

The wild-type tomato Ailsa Craig (AC^++^) and ripening inhibitor mutant tomato (*rin*) were cultivated under standard greenhouse conditions for routine management. The number of days after pollination (dpa) and fruit colour were used as the criteria for judging ripening period. AC fruits were harvested at MG (ripe green, 33 DPA, green and shiny, no obvious colour change) and 4 days after Breaker (B + 4, about 42 DPA, the fruit changed from green to yellow, defined as Breaker). The *rin* mutant fruits were harvested at the B + 4 stage. All plant samples were immediately frozen with liquid nitrogen and stored at −80 °C.

### 4.2. Protein Extraction and Trypsin Digestion

Total proteins were extracted from tomato fruits, as described previously [68]. Appropriate amounts of the MG- and B4-stage tissue samples were placed into a liquid nitrogen pre-cooled mortar and ground into powder. Four volumes of lysis buffer (8 M urea, 1% Triton X-100, 2 mM EDTA, 10 mM dithiothreitol, and 1% protease inhibitor) were added to each set of samples. After ultrasonic lysis, samples were centrifuged at 20,000× *g*, 4 °C for 10 min, then 20% trichloroacetic acid was added to the supernatant, and the mixture was allowed to stand at 4 °C for two hours. Next, centrifugation was conducted at 12,000× *g* and 4 °C for three minutes, after which the supernatant was discarded and the pellet was washed three times with pre-chilled acetone. The pellet was resuspended in 8-M urea and protein concentrations were determined with a BCA kit, according to the manufacturer instructions (Beyotime, Shanghai, China).

### 4.3. TMT Labeling and Enrichment of Phosphorylated Peptides

The protein solution was reduced with 5 mM of dithiothreitol at 56 °C for 30 min, and incubated with 11 mM iodoacetamide in the dark for 15 min at 25 °C. Finally, the resulting sample was diluted to less than 2 M, trypsin was added at a mass ratio of 1:50 (trypsin: protein) and the sample was digested overnight at 37 °C. Next, trypsin was added at a mass ratio of 1:100 (trypsin: protein), and the sample was digested for 4 h. After trypsinization, the peptides were desalted using Strata X C18 SPE column (Phenomenex, Torrance, California, USA) and vacuum-dried. Then the recombinant peptides were processed in 0.5 M TEAB, according to the manufacturer TMT kit protocol. The peptides were resuscitated with 500 mL loading buffer (2% trifluoroacetic acid/1% glutamate/65% acetonitrile). Then, TiO_2_ beads were added and the solution was incubated at 25 °C for 30 min. The centrifuged sample was washed twice with elution buffer I (0.5% trifluoroacetic acid/65% acetonitrile) and elution buffer II (0.1% trifluoroacetic acid/65% acetonitrile). The phosphopeptides were eluted with elution buffer I (50% acetonitrile/0.3 m NH4OH) and elution buffer II (60% acetonitrile/0.5 m NH4OH). The eluted peptides were lyophilized and stored at −20 °C.

### 4.4. HPLC Fractionation and LC-MS/MS Analysis

The HPLC method was used to separate tryptic peptides, using a C 18 column (length 250 mm, 5 μm particles, 4.6 mm ID, Agilent 300 Extend, Beijing, China). In short, a gradient of 8%-to-32% acetonitrile (pH 9.0) was utilized to separate the peptides into 60 fractions over 60 min. After being dissolved in solvent A (0.1% formic acid), a reversed-phase analysis column (75 μm i.d, 15 cm long) was prepared. The gradient of solvent B (98% acetonitrile and 0.1% formic acid) was increased from 6% to 23% over 26 min, then increased from 23% to 35% over 8 min, then to 80% over 3 min, and finally kept at 80% for an additional 3 min (flow rate of 400 nL/min, Easy-NLC 1000 UPLC). The peptides were analysed by MS/MS tandem mass spectrometry with NSI source, couple Q Exactive^TM^ Plus (Thermo, Shanghai, China) and UPLC online. The electrospray voltage was 2.0 kV. Full-scan M/Z scans ranged from 350 to 1800, and orbitrap detected complete peptides at a resolution of 70,000. Then the polypeptide with NCE setting to 28 was used for MS/MS. The fragment was detected at a resolution of 17,500. AGC is set to 50,000. The first mass was 100 m/z.

### 4.5. Protein Identification and Functional Annotation

MS/MS data was processed through Maxquant search engine (http://www.maxquant.org (v.1.5.2.8), accessed on 20 October 2021). The Proteome Solanum Lycopersicum (tomato) database was used for tandem mass spectrometry search. Firstly, the mass tolerance of the searched precursor ion was 20 PPM, the tolerance of the main search precursor ion was 5 PPM and the mass tolerance of the fragment ion was 0.02 Da. Carbamidomethyl on Cys was specified as fixed modification and oxidation on Met was specified as variable modifications. Adjusted FDR was less than 1% and the lowest score of peptides was greater than 40.

UniProt and GO databases were used to classify all the identified proteins and to clarify their biological processes and molecular functions [69]. The sequences of differentially displayed proteins were extracted and compared with the COG database [70]. The KEGG database was used to examine the protein pathways. The protein structures domain functions were identified by InterProScan (http://www.ebi.ac.uk/interpro/, accessed on 20 October 2021). Wolfpsort (https://wolfpsort.hgc.jp/, accessed on 20 October 2021) was used to predict subcellular localization. A *p*-value less than 0.05 was considered significant.

### 4.6. Motif Analysis

The Motif-X software was employed to analyse the motif characteristics of the modified sites. The peptide sequences consisting of six amino acids upstream and six amino acids downstream of all the identified modification sites were analysed. If the number of peptides containing a characteristic sequence was greater than 20 and its *p*-value less than 0.000001, it was considered to be a modified peptide motif.

### 4.7. Functional Enrichment

To detect enrichment of the differentially expressed proteins, the GO and KEGG database were used in the enrichment analyses. A two-tailed Fisher test was used to accurately detect the enrichments of all differentially expressed proteins. If their *p*-values were less than 0.05, they were considered significantly different.

### 4.8. Analysis of Kinases and Phosphatases

The tomato protein kinase database, iTAK, was used to analyse the detected kinases [71]. The phosphatases were obtained from the proteome and phosphoproteome data.

### 4.9. Prediction of Kinase Substrates, Kinase Activities and Kinase-Substrate Regulatory Networks

The GPS 5.0 software [72] was used to predict kinase-substrate regulation. The corresponding kinase proteins in the kinase family was obtained by comparison with the kinase sequence in the IEKPD2.0 database [73]. PPI information was used to filter potential false positive results. The “medium” threshold setting was selected in GPS 5.0.

It is accepted that changes in the phosphorylation level of a substrate site reflect the kinase regulatory status. The GSEA [74] method was adopted to predict kinase activities. The normalized enrichment score (NES) of the enrichment results was used as the kinase-activity score. The kinase was predicted as positive if the predominant change of substrates was an increase in phosphorylation and vice versa.

One kinase can regulate multiple substrates and a phosphorylation site may be regulated by more than one kinase. According to the complicated regulatory relationships, for each compare group, kinases predicted as positive or negative activity and significantly differential expressed phosphorylation sites were used to constructed kinase-substrate regulatory network.

### 4.10. Protein Interaction Analysis of the Proteome and the Phosphoproteome

The differential-expression and modified protein data obtained from the different groups in the proteome and phosphoproteome were combined with the STRING database version 11.0 (https://string-db.org/, accessed on 20 October 2021) for the analysis of protein-protein interactions. Then, the R package “NetworkD3” was used to visualize the PPI network. In order to show the protein-protein interaction relationships, the 50 most closely interacting proteins were selected and their protein interaction network was mapped in Cytoscape (https://cytoscape.org/, accessed on 20 October 2021).

### 4.11. Statistical Analysis

Statistical analysis was performed using SPSS 18.0 (SPSS, Chicago, IL). ANOVAs were used to analyze the data, and means were compared by the student’s *t* test. If *p* < 0.05, a difference was considered significant.

## Data Availability

The mass spectrometry proteomics and phosphoproteomics data generated from this study have been deposited to the ProteomeXchange Consortium via the PRIDE [75] partner repository with the dataset identifier PXD027844.

## Supplementary Materials

The following are available online at https://www.mdpi.com/article/10.3390/ijms222111782/s1.
