# Supplementary material for: Novel Translational and Phosphorylation Modification Regulation Mechanisms of Tomato (Solanum lycopersicum) Fruit Ripening Revealed by Integrative Proteomics and Phosphoproteomics
Source: Int J Mol Sci. 2021 Oct 29;22(21):11782. doi: 10.3390/ijms222111782 (PMC8584006; doi:10.3390/ijms222111782)
Supplement: Supplementary file 1 [file ijms-22-11782-s001.zip › Supplementary Figures.pdf]

SUPPLEMENTARY INFORMATION (SI)

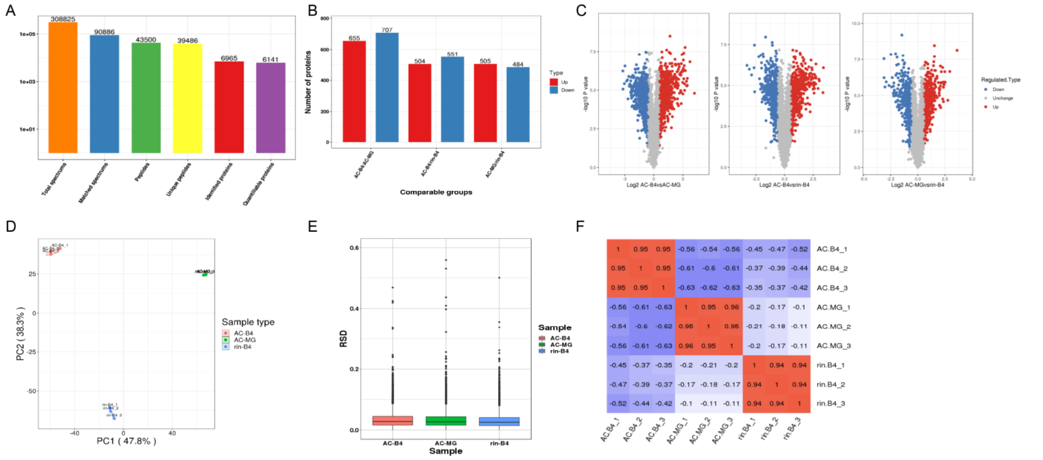

Figure S1. Overview of proteomics results. A. Basic statistical figure of MS results; B. Histogram of the number distribution of differentially expressed proteins in different comparison groups; C. Volcano plot of differentially expressed proteins; D. Two-dimensional scatter plot of PCA (principal component analysis) distribution of all samples using quantified proteins; E. Box plot of RSD (Relative Standard Deviation) distribution of repeated samples using quantified proteins; F. Heatmap of Pearson correlation coefficients from all quantified proteins between each pair of samples.

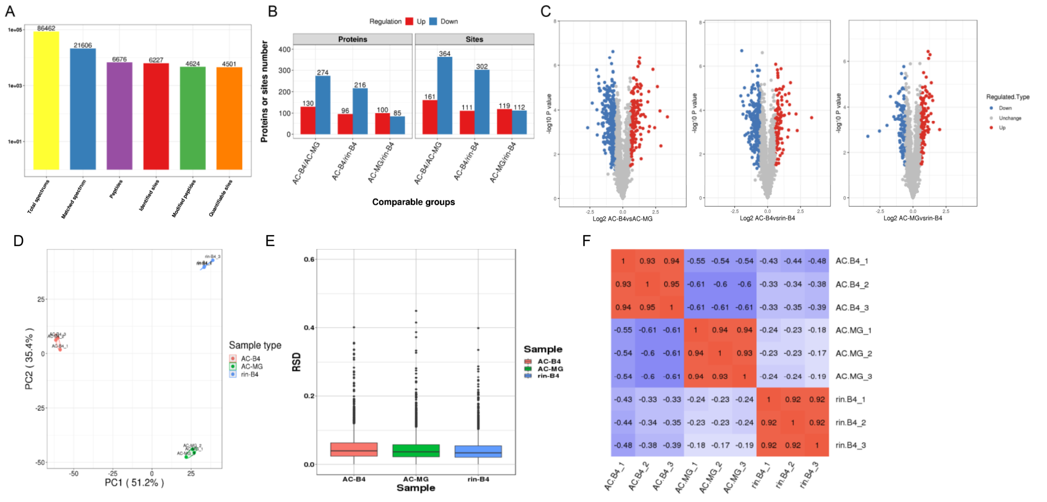

Figure S2. Overview of Phosphoproteomics results. A.Basic statistical figure of MS results; B. Histogram of the number distribution of differentially expressed proteins and modification sites in different comparison groups; C. Volcano plot of differentially expressed modification sites; D. Two-dimensional scatter plot of PCA (principal component analysis) distribution of all samples using quantified proteins; E. Box plot of RSD (Relative Standard Deviation) distribution of repeated samples using quantified proteins; F. Heatmap of Pearson correlation coefficients from all quantified proteins between each pair of samples.

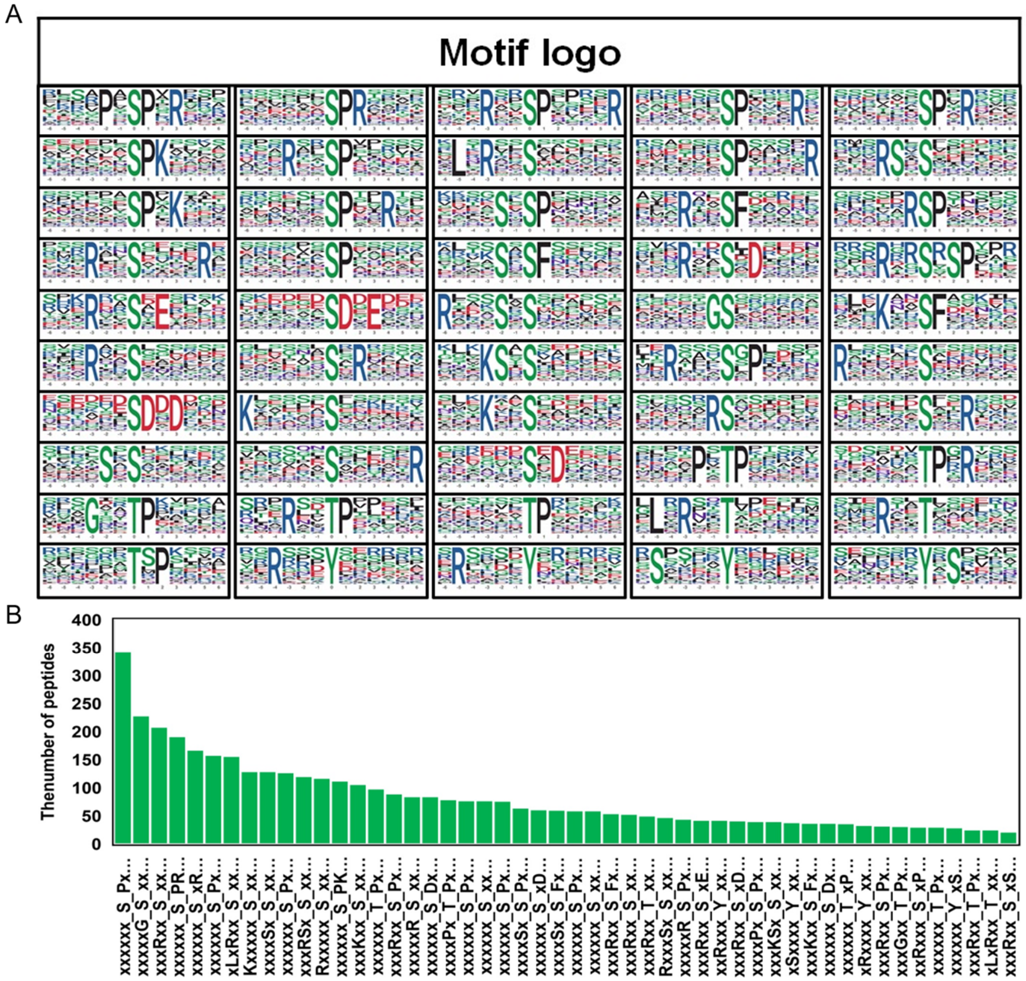

Figure S3. Analysis of phosphorylation sites. (A) Sequence motif analysis of phosphorylation sites. (B) The number of identified peptides containing phosphorylation sites in each motif.

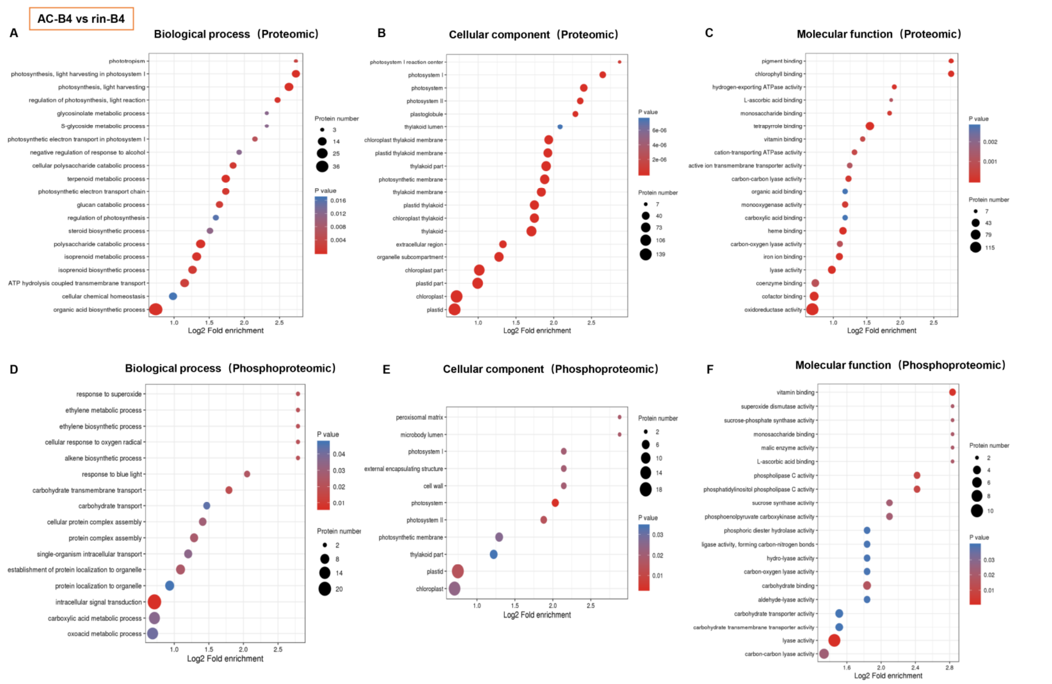

Figure S4. Bubble chart of enrichment distribution of DEPs and DEPPs in GO functional classification (AC-B4 vs rin-B4). Bubble chart of enrichment distribution of differentially expressed proteins (A, B and C) and differentially phosphorylated proteins (D, E and F) in GO functional classification. (AC-B4 vs rin-B4).

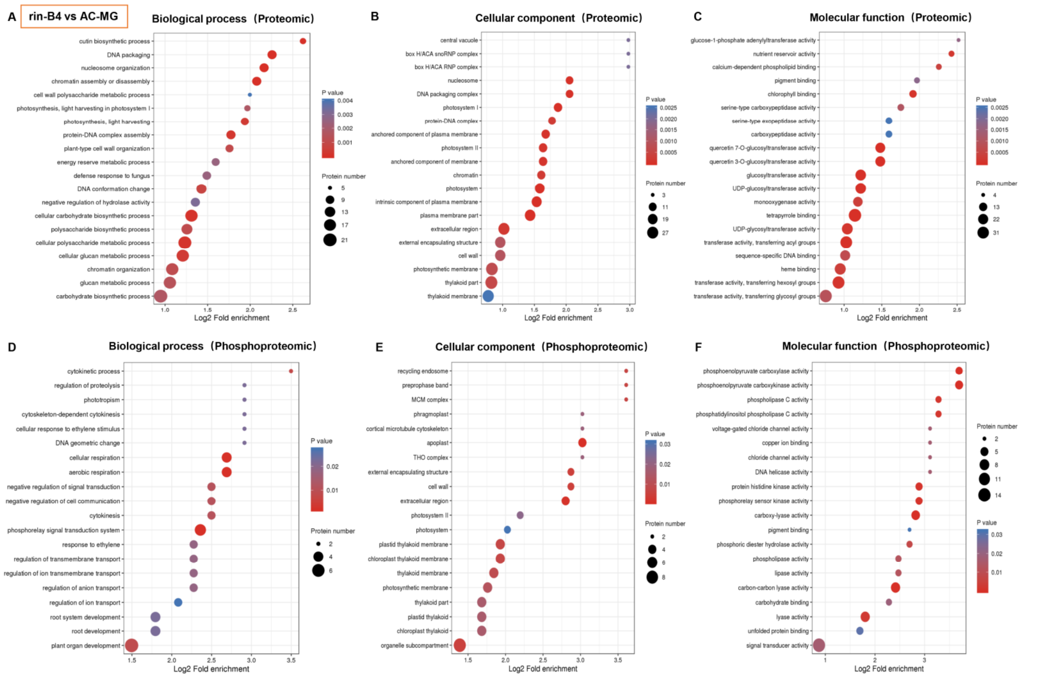

Figure S5. Bubble chart of enrichment distribution of DEPs and DEPPs in GO functional classification (rin-B4 vs AC-MG). Bubble chart of enrichment distribution of differentially expressed proteins (A, B and C) and differentially phosphorylated proteins (D, E and F) in GO functional classification.

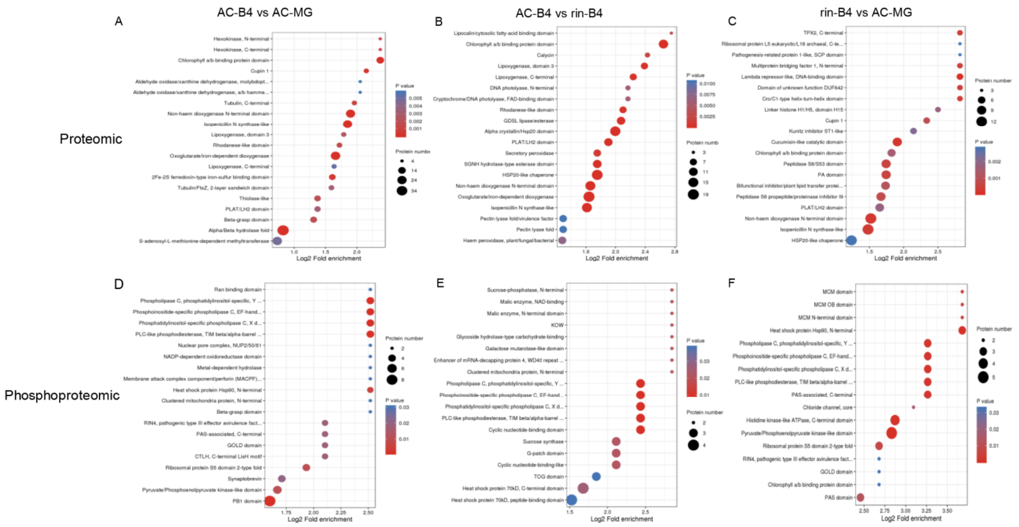

Figure S6. Protein domain enrichment bubble chart of distribution of DEPs and DEPPs. Protein domain enrichment bubble chart of distribution of differentially expressed proteins (A,B and C) and proteins corresponding to differentially expressed modification sites (D, E and F) in different comparison groups.

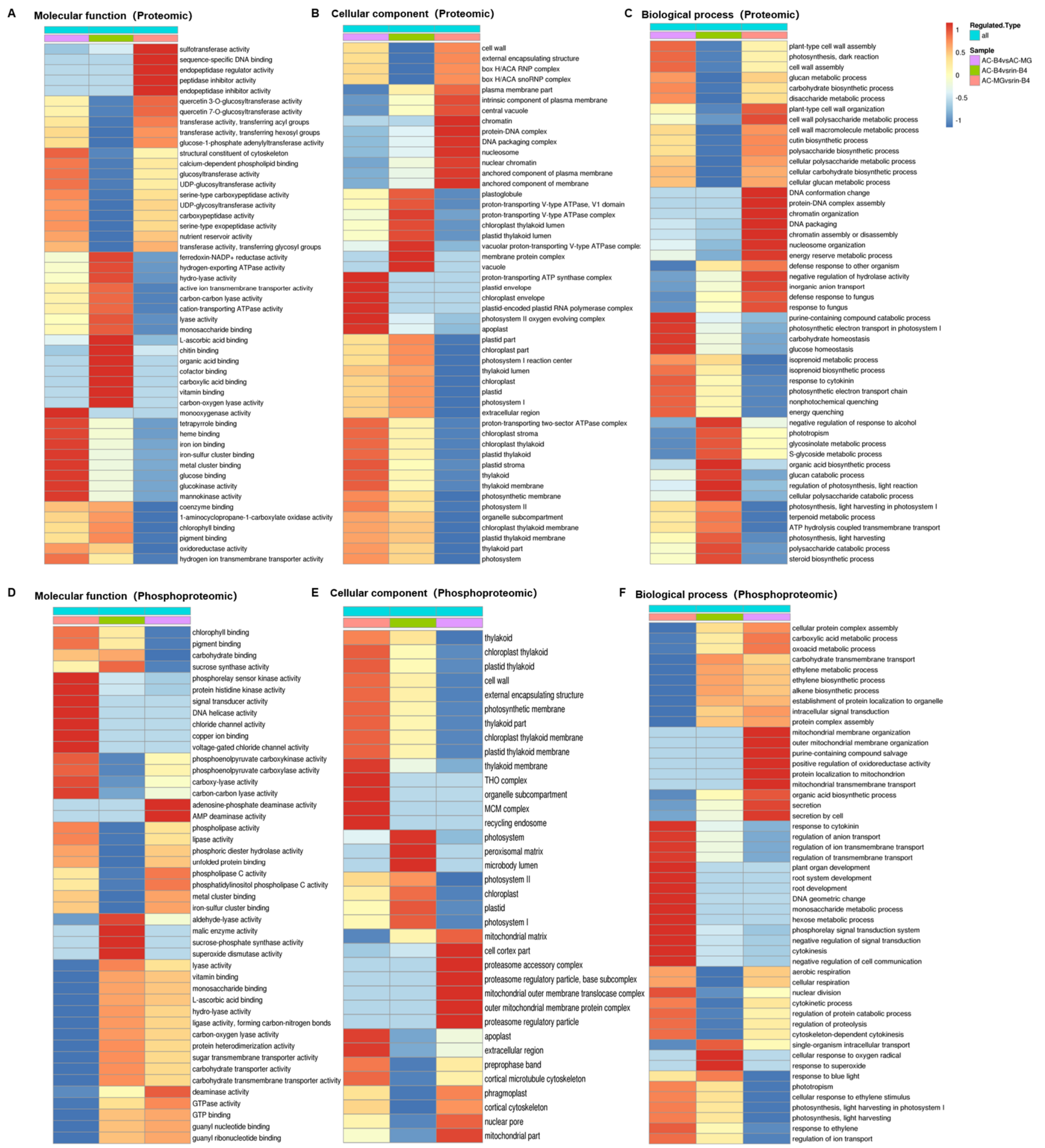

Figure S7. A comprehensive heatmap for cluster analysis of the enrichment patterns of GO functional categories in proteomics (A, B and C) and phosphoproteomics (D, E and F). (Molecular function, Cellular component and Biological process).

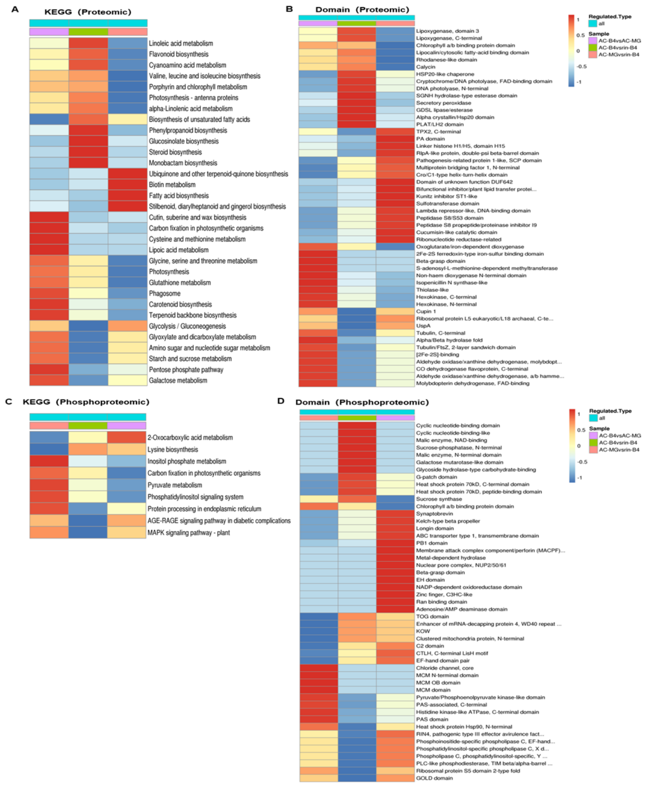

Figure S8. A comprehensive heatmap for cluster analysis of the enrichment patterns of KEGG pathways and protein domains in proteomics (A and B) and phosphoproteomics (C and D).

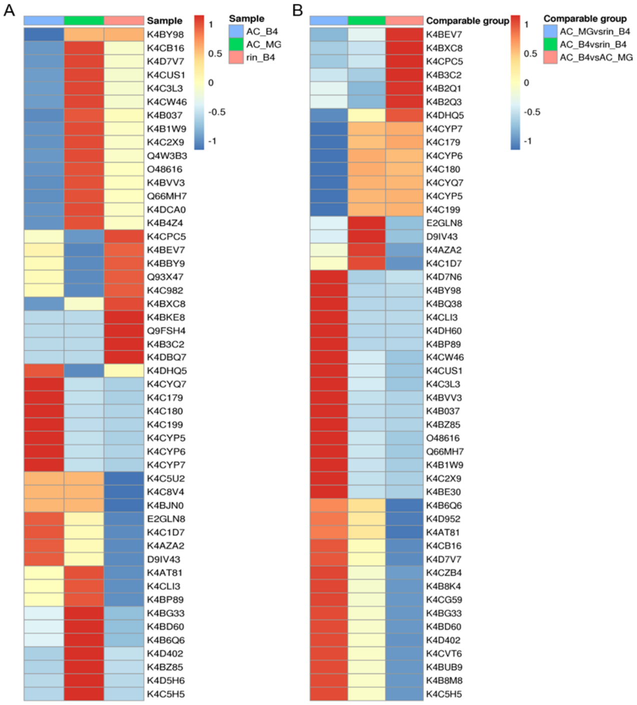

Figure S9. Clustering heat map of phosphokinase activity. A. Different samples; B. Different comparison groups. Red represents activation status, blue represents inhibition status.

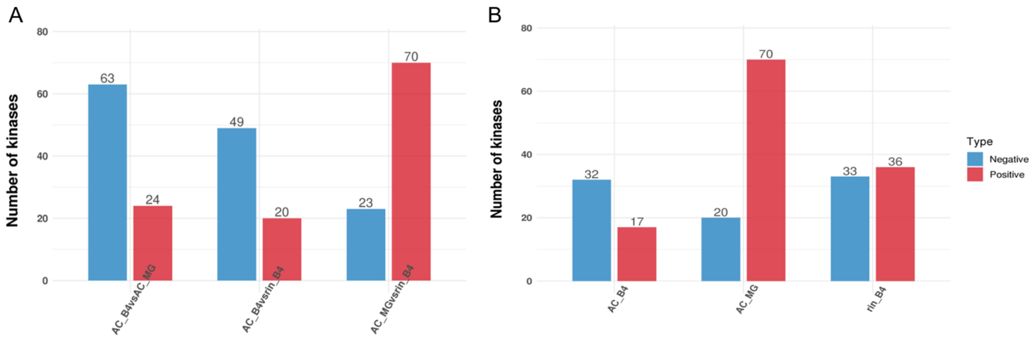

Figure S10. Phosphokinase activity statistics. The horizontal axis is the name of the comparison groups (A) and samples (B), and the vertical axis is the number of kinases. Red represents kinase activity tends to be activated, and blue represents kinase activity tends to be inhibited.
